# Endogenous Amplification of Apoptosis via p53 Regulation using a Cascade Nanocatalytic Medicine

**DOI:** 10.1002/advs.202520367

**Published:** 2026-02-11

**Authors:** Tan Wu, Xiaoyue Xu, Dan Xu, Hui Zhang, Nan Wang, Yang Yang, Qian Chen, Shunjie Chen, Cheng Li

**Affiliations:** ^1^ Department of Anesthesiology and Perioperative medicine, Shanghai Key Laboratory of Anesthesiology and Brain Functional Modulation, Clinical Research Center For Anesthesiology and Perioperative Medicine, Translational Research Institute of Brain and Brain‐Like Intelligence, School of Medicine, Shanghai Fourth People's Hospital Tongji University Shanghai China; ^2^ Department of General Surgery Xinhua Hospital Affiliated to Shanghai Jiao Tong University School of Medicine Shanghai China; ^3^ Department of Nephrology, School of Medicine, Shanghai Fourth People's Hospital Tongji University Shanghai China

**Keywords:** colorectal cancer, DNA, MOF, nanocatalysis, p53 protein, peroxynitrite

## Abstract

Nanocatalytic therapy is an emerging strategy that leverages in situ catalytic reactions within the tumor microenvironment to convert endogenous substrates into cytotoxic species, achieving spatially confined cancer cell killing with reduced systemic toxicity. However, the lack of durable, DNA‐focused cytotoxic mechanisms hampers the translational efficacy of nanocatalytic therapy. Herein, we proposed a cascade nanocatalysis‐mediated strategy for endogenous amplification of apoptosis, achieved by an engineered metal organic framework nanomedicine (MOF‐Au‐L‐Arginine, abbreviated as MAL). The MOF serves both as a nanocatalyst and as a carrier for L‐Arginine (L‐Arg), while embedded Au nanoparticles enhance nanocatalyst reactivity. Subsequently, MOF catalyzes the generation of hydroxyl radicals (•OH) and superoxide anions (O_2_^•−^), and then the O_2_^•−^ undergo a cascade reaction with NO released from L‐Arg, generating highly cytotoxic peroxynitrite (ONOO–), which has greater cytotoxicity to tumor cells, can induce extensive DNA damage, and simultaneously impair DNA repair and disrupt the cell cycle. Genome‐wide RNA sequencing reveals MAL can activate the p53 pathway, thereby regulating apoptosis related proteins. In addition, MAL reduces mitochondrial membrane potential and promotes mitochondrial‐mediated apoptosis through the BAX/Bcl‐2/caspase‐3 axis, further amplifying endogenous apoptosis in tumor cells. In vivo, MAL effectively inhibits tumor growth with favorable biocompatibility.

## Graphical abstract

An engineered MOF nanomedicine (MAL) triggers cascade nanocatalysis in tumors, converting H_2_O_2_ and L‐Arg into cytotoxic peroxynitrite. This induces DNA damage, activates the p53 pathway, and amplifies mitochondrial apoptosis via the BAX/Bcl‐2/Caspase‐3 axis, effectively inhibiting colorectal cancer growth with high biocompatibility.

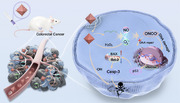

## 1 Introduction

By using the intrinsic chemical characteristics of nanomaterials to catalyze processes inside the tumor microenvironment, nanocatalytic therapy has become a viable cancer treatment approach [1]. Unlike conventional therapies, which often suffer from systemic toxicity and limited specificity, nanocatalytic therapies harness localized catalytic processes to selectively induce cytotoxic effects in tumor cells. This approach capitalizes on the tumor's biochemical landscape, including its abundant endogenous substrates such as hydrogen peroxide, which are utilized to generate reactive oxygen species (ROS) and other cytotoxic agents. Iron based metal organic frameworks are widely used as catalysts and drug carriers for Fenton reactions due to their large specific surface area and good biosafety [2, 3, 4, 5, 6]. However, the catalytic efficiency of a single MOF has limitations, and the ROS generated by its catalysis is still not ideal for treating tumors [7, 8, 9]. By modifying MOF with inorganic nanoparticles, organic compounds, and other active substances [10, 11, 12, 13, 14, 15, 16, 17], their catalytic efficiency can be significantly improved, enhancing the therapeutic effect on tumors. In particular, prior investigations have indicated that gold nanoparticles possess peroxidase activity and are capable of catalyzing the breakdown of H_2_O_2_ into •OH. It is anticipated that using gold nanoparticles supported on different substrates as catalysts will encourage the Fenton reaction, enhancing the catalytic activity of MOF and tumor‐treating potential [18, 19, 20]. Therefore, combining MOF with gold nanoparticles is a new strategy to improve the catalytic efficiency of MOF.

Although nanocatalytic therapy has many advantages, its efficacy is greatly reduced due to the short diffusion distance of ROS, low instantaneous lifespan and easy clearance by tumor associated antioxidant enzymes, the intracellular DNA repair mechanism further limits the efficacy of CDT [21, 22, 23, 24, 25]. Recent studies have shown that O_2_^•−^ can react with NO to form ONOO**^−^**. Due to its potent oxidation and nitrification properties, ONOO**^−^** is essential for controlling both physiological and pathological processes, and can participate in the regulation of various diseases [26, 27, 28, 29]. Compared with ROS such as •OH and O_2_^•−^, ONOO**^−^** has a longer lifespan and diffusion distance, is difficult to remove, and has higher cytotoxicity [30, 31]. In addition, ONOO**^−^** can oxidize DNA and may cause DNA damage by attacking the sugar phosphate backbone [32, 33, 34, 35]. p53 is a tumor suppressor that regulates DNA repair, cell apoptosis, and mediates a series of anti‐tumor proliferation processes [36, 37]. When DNA double strand breaks are detected, the ATM/CHK2/p53 axis of the cell is activated [38, 39]. The Bcl‐2 protein family is a key factor in mitochondrial apoptosis, and activation of p53 can further regulate Bcl‐2 family proteins, ultimately leading to mitochondrial dysfunction and activation of the apoptotic program, further triggering tumor cell apoptosis [40, 41, 42, 43, 44, 45, 46]. Therefore, it is anticipated that combining ONOO**^−^** mediated DNA damage and DNA repair inhibition with CDT therapy will alleviate the short‐term toxicity of ROS in CDT alone and offer a novel tumor therapeutic approach.

**SCHEME 1:**
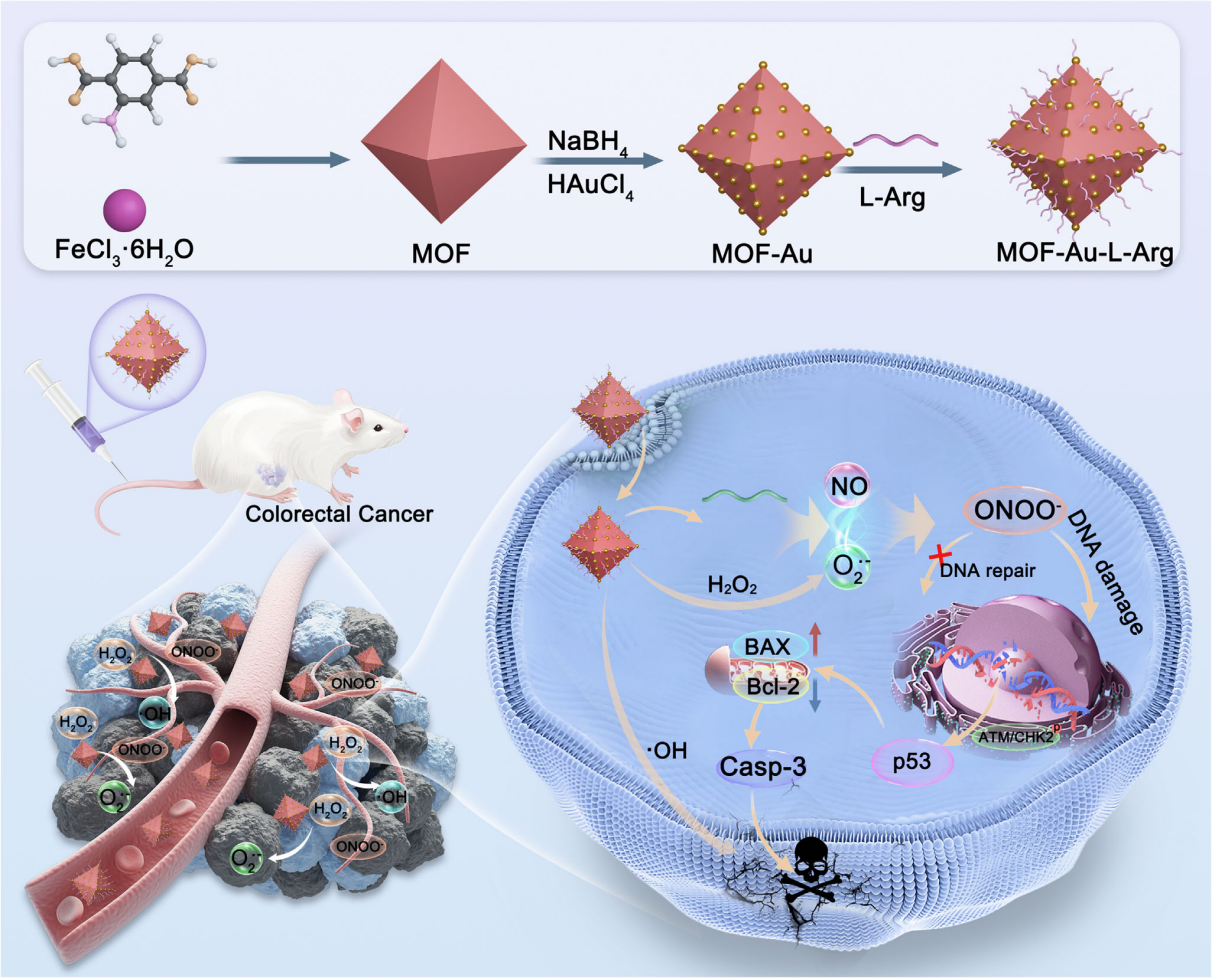
Diagrammatic illustration of the MAL synthesis process and its TME‐activated cancer nanocatalytic therapy mechanism. MAL catalyzes H_2_O_2_ to produce •OH and O_2_^•−^. NO and O_2_^•−^ reactions further produce more ONOO**^−^**,. which induces DNA damage and inhibits DNA repair. ATM/CHK2/p53 senses DNA damage, reduces mitochondrial membrane potential, and activates downstream apoptotic proteins BAX/Bcl‐2/Casp‐3, further amplifying endogenous apoptosis in tumor cells.

In this study, we propose a novel engineered nanomedicine (MOF‐Au‐L‐Arg, abbreviated as MAL) to enhance nanocatalytic therapy through a cascade reaction that generates ONOO**^−^**. By incorporating gold nanoparticles into a metal‐organic framework (NH2‐MIL‐88B (Fe)) and loading it with L‐Arg, a nitric oxide donor, MAL significantly amplifies the nanocatalytic reaction, generating higher concentrations of •OH and O_2_^•−^. The generated O_2_^•−^ then reacts with NO released from L‐Arg to form ONOO**^−^**, which induces DNA damage and activates the ataxia‐ telangiectasia mutated/checkpoint kinase 2/p53 (ATM/CHK2/p53) signaling pathway. By reducing the mitochondrial membrane potential, downregulating the anti‐apoptotic protein Bcl‐2 and upregulating the pro‐apoptotic protein BAX, ultimately activating the caspase‐3 apoptotic protein to induce mitochondrial‐mediated apoptosis. This cascade process increases endogenous apoptosis of tumor cells. In vivo, the MAL nanomedicine demonstrates enhanced therapeutic efficacy, significantly inhibiting tumor growth in a colorectal cancer mouse model, making it a promising strategy for improving the outcomes of nanocatalytic cancer therapies (Scheme 1).

## 2 Results and Discussion

### 2.1 Synthesis and Characterization of MAL

**FIGURE 1:**
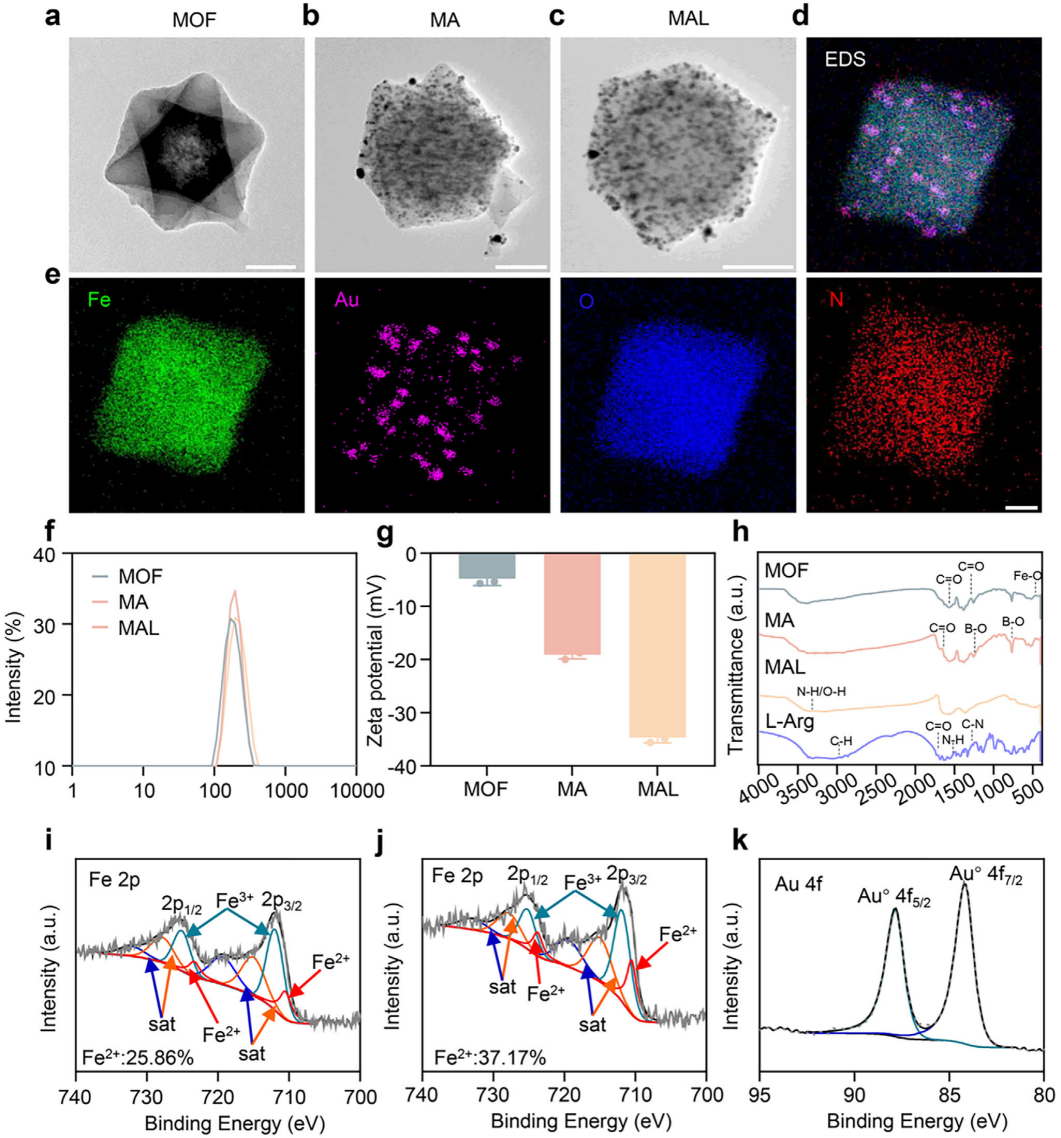
Synthesis and characterization of MAL. TEM images of a) MOF, b) MA, and c) MAL. Scale bar: 100 nm. d,e) EDS and elemental mapping of Fe, Au, O, and N of MAL. Scale bar: 50 nm. f) Hydrodynamic size distribution of MOF, MA, and MAL measured by DLS. g) Zeta potential of MOF, MA, and MAL (n = 3 independent samples, mean ± SD). h) FTIR spectra of MOF, MA, and MAL.i) XPS spectra of Fe 2p in MA. XPS spectrum of j) Fe 2p and k) Au 4f in MAL.

Firstly, according to previous literature reports, NH_2_‐MIL‐88B (Fe), also known as a Metal‐Organic Framework (MOF), was synthesized [47]. The transmission electron microscopy (TEM) and scanning electron microscope (SEM) results confirmed that the MOF exhibited a uniformly sized octahedral structure (Figure 1a; Figure S1). Next, gold nanoparticles were loaded into MOF through redox reactions to form MOF‐Au (MA) (Figure 1b) [48]. Subsequently, L‐Arg as a NO donor was loaded into MA to form nanoparticle MAL (Figure 1c). The dynamic light scattering (DLS) analysis showed that the hydrodynamic size of MAL was approximately 198.9 nm (Figure 1f). The Zeta potential also changed from −4.77 to −34.65 mV (Figure 1g). The energy dispersive X‐ray spectroscopy (EDS) image and elemental mapping of MAL confirmed the successful loading of gold element (Figure 1d,e). Meanwhile, we further characterized MAL by UV–vis absorption spectroscopy and Fourier transform infrared spectroscopy (FTIR) (Figure 1h; Figure S2). The absorption peak of sample at 3380 cm^−1^ has a much higher relative intensity when L‐Arg is added, indicating that L‐Arg was successfully loaded and formed strong hydrogen bonding interactions with the organic ligands on the MOF surface. The L‐Arg reagent kit and standard curve were used to confirm that the loading efficiency of L‐Arg was 22% (Figure S3) [49].

In addition, X‐ray photoelectron spectroscopy (XPS) was also used to reveal the elements of MA and MAL (Figure 1i–k; Figure S4). XPS analysis revealed a notable increase in the Fe^2+^/Fe^3+^ ratio in MAL compared to MA. This could be attributed to the electron‐donating character of the amino and guanidinyl groups in L‐arginine, which coordinate with the MOF, increasing local electron density at Fe sites and stabilizing the Fe^2+^ state. This modulation enhances the density of active sites for H_2_O_2_ activation, directly contributing to the improved catalytic performance of MAL. The electron binding energy positions of Au element at 84.15 and 87.83 eV correspond to 4f 7/2 and 4f 5/2 of Au^0^, this indicates that the gold element exists in the form of elemental metal [50]. Respectively, further demonstrating the successful loading of gold nanoparticles.

### 2.2 Extracellular Catalytic Performance

**FIGURE 2:**
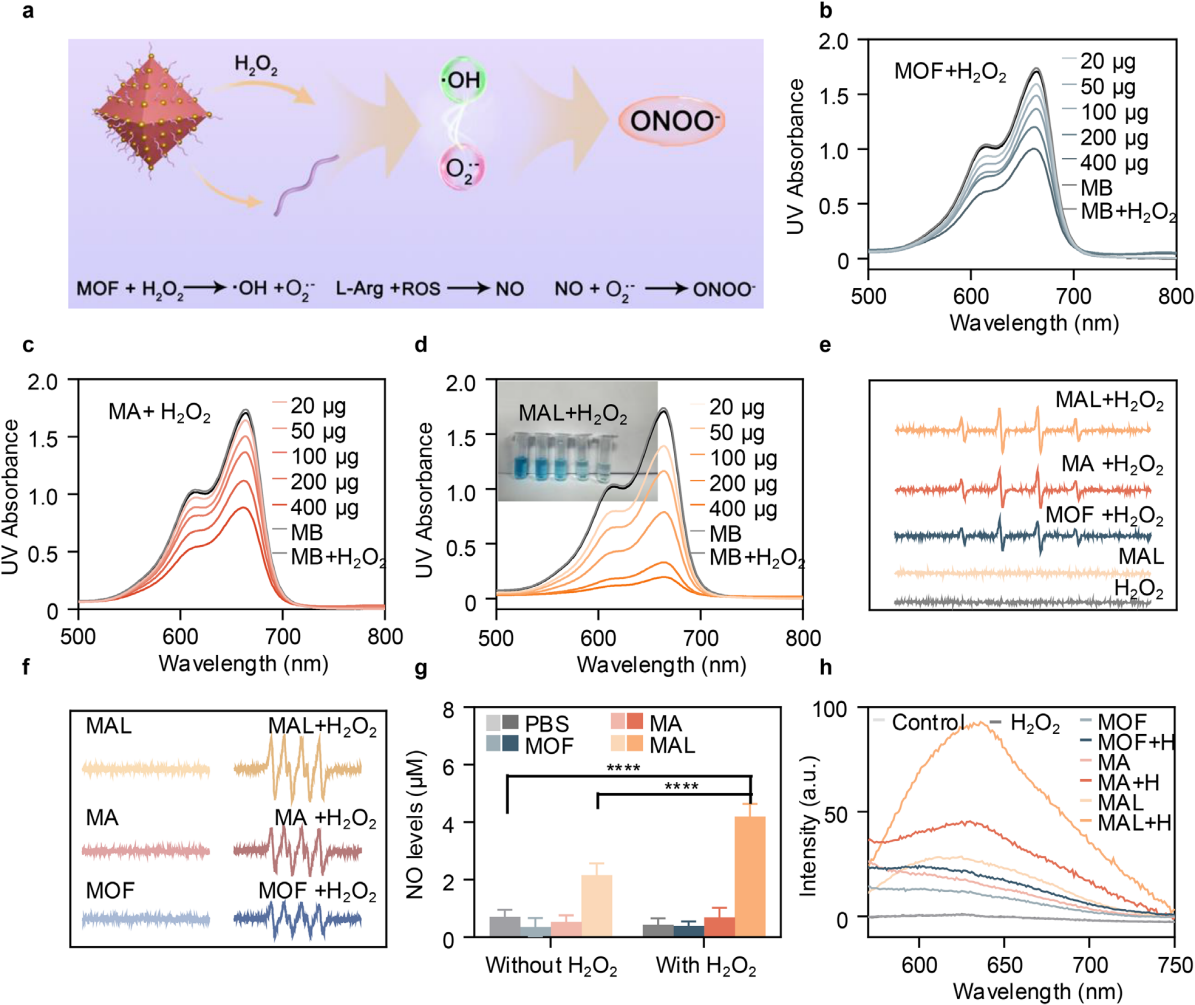
The enzymatic catalytic activity of MAL. a) Schematic diagram of the mechanism for MOF‐Ag‐L‐Arg to generate •OH, O_2_^•−^, NO, and ONOO**^−^**. UV–vis absorption spectra of MB after reaction with different concentrations of b) MOF+H_2_O_2_, c) MA+H_2_O_2_, and, d) MAL+H_2_O_2_ (inset: centrifuge tubes containing the corresponding mixture). e) ESR spectra of •OH trapped by DMPO under H_2_O_2_. f) ESR spectra of O_2_^•−^ trapped by DMPO under H_2_O_2_. g) NO concentration produced by PBS, MOF, MA, and MAL reacting with H_2_O_2_. h) ONOO**^−^** generation of different treatments with/without H_2_O_2_. Data were shown as mean ± SD (n = 3). ^*^ *p* < 0.05, ^**^ *p* < 0.01, ^***^ *p* < 0.001, ^****^ *p* < 0.0001.

First, the catalytic mechanism diagram of MAL catalyzing the generation of ROS in the tumor microenvironment, followed by a series of cascading reactions to produce ONOO**^−^** was demonstrated (Figure 2a). Next, to examine the potential for accelerating the production of ROS with or without H_2_O_2_. Methylene Blue (MB) was selected as the probe for the experiment and the ability of different groups of nanoparticles to produce ROS [51]. Without the absence of H_2_O_2_, even with an increase in drug concentration, MB only showed slight degradation in each group (Figure S5). With the increase of drug concentration and the presence of H_2_O_2_, as ROS production rises, the UV–vis characteristic absorption peak of MB at 664 nm steadily declines. When the concentration of MAL+H_2_O_2_ group increased to 400 µg, the color of MB almost became colorless, and the characteristic absorption peak of UV also showed a cliff like decrease. However, even though the absorption peak of MA+H_2_O_2_ and MOF+H_2_O_2_ decreased, the amplitude was much lower than that of MAL+H_2_O_2_. This indicates that the ROS produced by MAL is significantly higher than that of MOF+H_2_O_2_ and MA+H_2_O_2_ groups (Figure 2b–d). This is attributed to the presence of gold nanoparticles and L‐Arg, which enhances the catalytic ability of MAL. Specifically, according to previous reports, gold nanoparticles have peroxidase activity and the ability to accelerate the conversion of H_2_O_2_ to ROS. In addition, L‐Arg may benefit from inducing the formation of a large number of oxygen vacancies (OV), thereby improving the adsorption and conversion of H_2_O_2_ and enhancing the catalytic performance of MOF [52, 53]. To further confirm the generation of ROS, 5,5'dimethylpyrroline 1‐oxide (DMPO) was used for validation [54]. When only H_2_O_2_ or pure drugs are present, there are almost no characteristic peaks representing •OH (Figure S6). When H_2_O_2_ was added, MAL+H_2_O_2_ group produced the strongest •OH and O_2_^•−^ signals, which also indicates that the MAL group produced a large amount of ROS (Figure 2e,f). Then, the detailed catalytic performance of MAL under different concentrations of H_2_O_2_ was studied using Michaelis–Menten kinetics. After recording the relative absorbance data of phase MB and calculating it, Michaelis–Menten curve and Lineweaver Burk plot were plotted, which are the linear reciprocal plots of Michaelis‐Menten equation. The results indicate that MAL has the highest reaction rate, The maximum velocity (V_max_), and the Michaelis constant (K_m_) were calculated to be 9.4 × 10^−6^ m min^−1^ and 0.084 mm (Figure S7). The K_m_ value, which reflects the enzyme‐substrate affinity, is the lowest for MAL among the tested materials, indicating its highest catalytic efficiency and strongest substrate binding, consistent with previously reported nanozyme studies [1]. Based on the above experimental results, we used density functional theory (DFT) calculations to simulate the Fenton reaction of nanomedicine models, in order to study their catalytic performance in more detail. The calculation results indicate that the MAL group has the lowest apparent energy barrier (−0.26 eV) and the strongest adsorption of hydrogen peroxide species, which is more favorable for the Fenton reaction (Figure S8) [55]. This is consistent with the results of Michaelis Menten kinetics, where the MAL group exhibits the strongest adsorption of H_2_O_2_. Therefore, MAL has higher catalytic performance and can achieve maximum catalytic activity at limited H_2_O_2_.

The generation of ROS will once again produce NO gas with L‐Arg, which can cascade with ROS to produce ONOO**^−^** with higher cytotoxicity, thereby improving the therapeutic effect on tumors [56]. Next, the Griess test kit was used to confirm MAL's capacity to generate NO in vitro. The signal of NO in the control group showed almost no change, while the MAL group produced an increasing concentration of NO signal after adding H_2_O_2_ (Figure S9). Subsequently, we verified whether the material could generate NO at the cellular level. It is evident that the MAL+H_2_O_2_ group produced the highest amount of NO (Figure 2g).

With that in mind, NO can combine with O_2_^•−^ to produce ONOO**^−^**, which poses a greater risk than ROS. As a result, ONOO**^−^** fluorescent probes were added to medication solutions of various groups. After incubation in the dark at 37°C for 2 h, the fluorescence signal generated by MAL+H_2_O_2_ was detected using a fluorescence spectrometer. A significant ONOO**^−^** fluorescence signal was seen at 600–650 nm (Figure 2h). This suggests that MAL has the ability to stimulate the production of ROS, which can then cascade with NO to create ONOO**^−^**.

### 2.3 Effect of Extracorporeal Therapy

**FIGURE 3:**
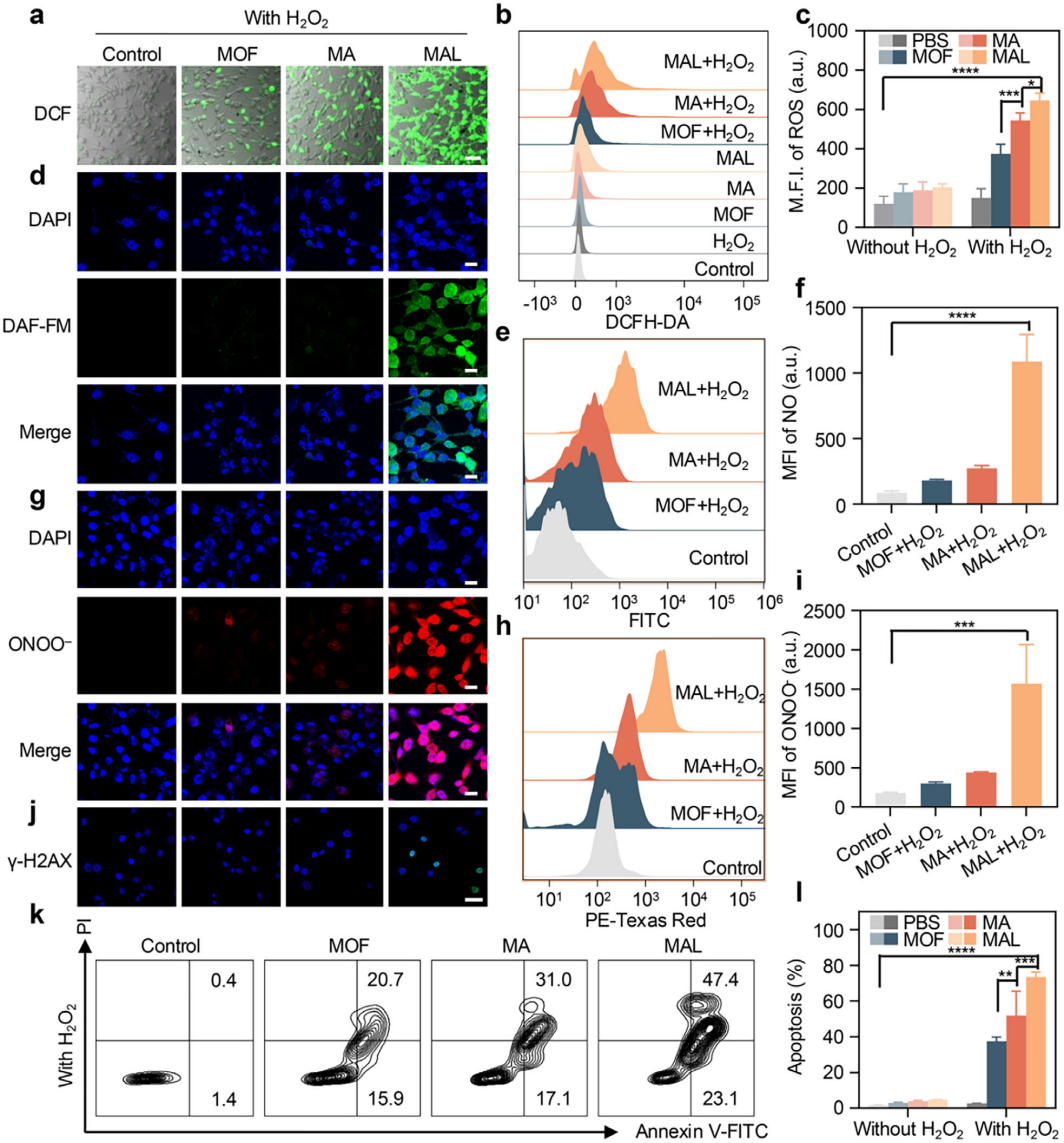
Assessment of the therapeutic effects of MAL in vitro. a) Merge plot of intracellular ROS production under different treatment conditions. Scale bar: 50 µm. b) Flow cytometry plot and c) quantitative analysis of the ROS level in CT26 cells (n = 3). d) Fluorescence pictures of CT26 cells labeled with DAF‐FM and 4′,6‐diamidino‐2‐phenylindole (DAPI) to detect intracellular NO. Scale bar: 20 µm. e) Flow cytometry analysis of DAF‐FM intensity to detect intracellular NO and f) the corresponding mean fluorescence intensity (MFI) (n = 3). g) CLSM images of ONOO**^−^**. Scale bar: 20 µm. h) Analysis of ONOO**^−^** intensity by flow cytometry and i) the corresponding MFI. j) γ‐H2AX foci of CT26 cells were detected by γ‐H2AX immunofluorescence after treatment. Scale bar: 20 µm. k) The apoptosis of CT26 cells was measured by flow cytometry. l) Quantification of apoptosis ratio (n = 3). Data were shown as mean ± SD (n = 3). ^*^ *p* < 0.05, ^**^ *p* < 0.01, ^***^ *p* < 0.001, ^****^ *p* < 0.0001.

First, 2',7'‐dichlorofluorescein was employed as a probe to confirm that CT26 cells produced ROS following various treatments. At the concentration used in our study, the control and H_2_O_2_‐only group did not generate significant DCF fluorescence, which is consistent with previous reports (Figure S10) [17]. After adding H_2_O_2_, the fluorescence intensity of MAL+H_2_O_2_ was noticeably more powerful than the other groups (Figure 3a). Subsequently, the production of ROS was further confirmed using flow cytometry, with results consistent with those obtained with confocal laser scanning microscopy (CLSM). The MAL+H_2_O_2_ group had the highest fluorescence intensity (Figure 3b,c), indicating that the nanocatalytic performance of MAL was optimal.

Subsequently, 3‐Amino,4‐aminomethyl‐2',7'‐difluorescein (DAF‐FM) was used as a tracer to detect NO and verify the production of intracellular NO. The CLSM results showed minimal changes in green fluorescence between the pure nanoparticles and the control group (Figure S11). In contrast, the addition of H_2_O_2_ induced significant green fluorescence in the MAL group (Figure 3d), indicating that L‐Arg reacts with ROS to produce NO. Subsequently, flow cytometry analysis further confirmed these findings, showing that compared to the other groups, the fluorescence intensity of MAL+H_2_O_2_ group was much greater. (Figure 3e,f), thereby further confirming the ability of MAL+H_2_O_2_ to produce NO.

Finally, to investigate whether ONOO**^−^** can be produced in CT26 cells, CLSM and flow cytometry analysis were performed using ONOO**^−^** fluorescent probes. In the absence of H_2_O_2_ and L‐Arg, only faint red fluorescence was seen in CT26 cells. After adding H_2_O_2_ and generating ROS with nanoparticles, strong red fluorescence was observed inside the cells (Figure 3g–i; Figure S12). This is due to the production of ROS and NO in CT26 cells. To further investigate the impact of MAL NPs on mitochondrial function, JC‐1 was used to detect mitochondrial membrane potential (MMP) after treatment to assess mitochondrial status (Figure S13) [57]. As expected, MAL+H_2_O_2_ induced the most effective mitochondrial damage, with the weakest red fluorescence and strongest green fluorescence, leading to the most significant depolarization of mitochondria and promotion of cell apoptosis. According to earlier research, oxidative damage brought on by ONOO**^−^** can also result in DNA damage, and γ‐H2AX is a biomarker of DNA damage. Therefore, the levels of γ‐H2AX in CT26 cells were investigated after treatment with different groups. The CLSM results showed that compared with the MOF+H_2_O_2_, MA+H_2_O_2_, and control groups, MAL+H_2_O_2_ produced significant green fluorescence, indicating that the DNA inside the cells was damaged, while the other groups hardly caused DNA damage. This is attributed to the cascade generation of ONOO**^−^** after loading L‐Arg (Figure 3j; Figure S14), which may be beneficial for combating tumor proliferation. To further investigate the DNA damage induced by MAL‐mediated ONOO^−^ formation, we employed the comet assay, a direct method for detecting DNA strand breaks. Notably, MAL+H_2_O_2_ group observed significant comet tails (Figure S15), demonstrating that MAL+H_2_O_2_ can cause DNA damage in cells, which is attributed to the presence of ONOO^−^.

Due to its excellent catalytic ability and ability to produce ONOO**^−^**, the efficiency of MAL in inhibiting tumor cells was further investigated. The Cell Counting Kit‐8 (CCK‐8) test kit was utilized to determine the cytotoxicity of nanoparticles. Compared to the MOF group, the toxicity of MA and MAL slightly increased, with a cell survival rate of over 70% even at a concentration of 100 µg (Figure S16a). Next, each group received the same amount of H_2_O_2_ to confirm the ability of nanoparticles to suppress tumor cells, and the results showed that CT26 cells were inhibited in a concentration gradient manner. The inhibition rate of MAL+H_2_O_2_ was notably more powerful than the other two groups (Figure S16b), which may be due to the generation of more ROS and highly toxic ONOO**^−^** by MAL+H_2_O_2_. In addition, we also investigated the effect of gold nanoparticles on tumor suppression at the cellular level. The results a indicate that the MA group has a higher tumor suppression effect compared to the ML group (Figure S17). Next, the viability of cells was studied using Calcein‐Acetylmethyl Ester/Propidium Iodide (Calcein‐AM/PI). It is evident that MAL+H_2_O_2_ exhibits strong red fluorescence and weak green fluorescence (Figure S18), demonstrating that MAL+H_2_O_2_ dramatically suppresses the development of tumor cells. Finally, the inhibitory effect of nanoparticles on cells was further evaluated through flow cytometry analysis. The simple administration group only showed a weak apoptosis rate (Figure S19). It is worth noting that after treatment with MAL+H_2_O_2_, the early and late apoptosis of CT26 cells were noticeably greater than those found in the MAL and MOF groups (Figure 3k,l), demonstrating that MAL induced ROS and ONOO**^−^** have potent cell killing ability.

### 2.4 In Vitro Evaluation of the Anticancer Mechanism of MAL

**FIGURE 4:**
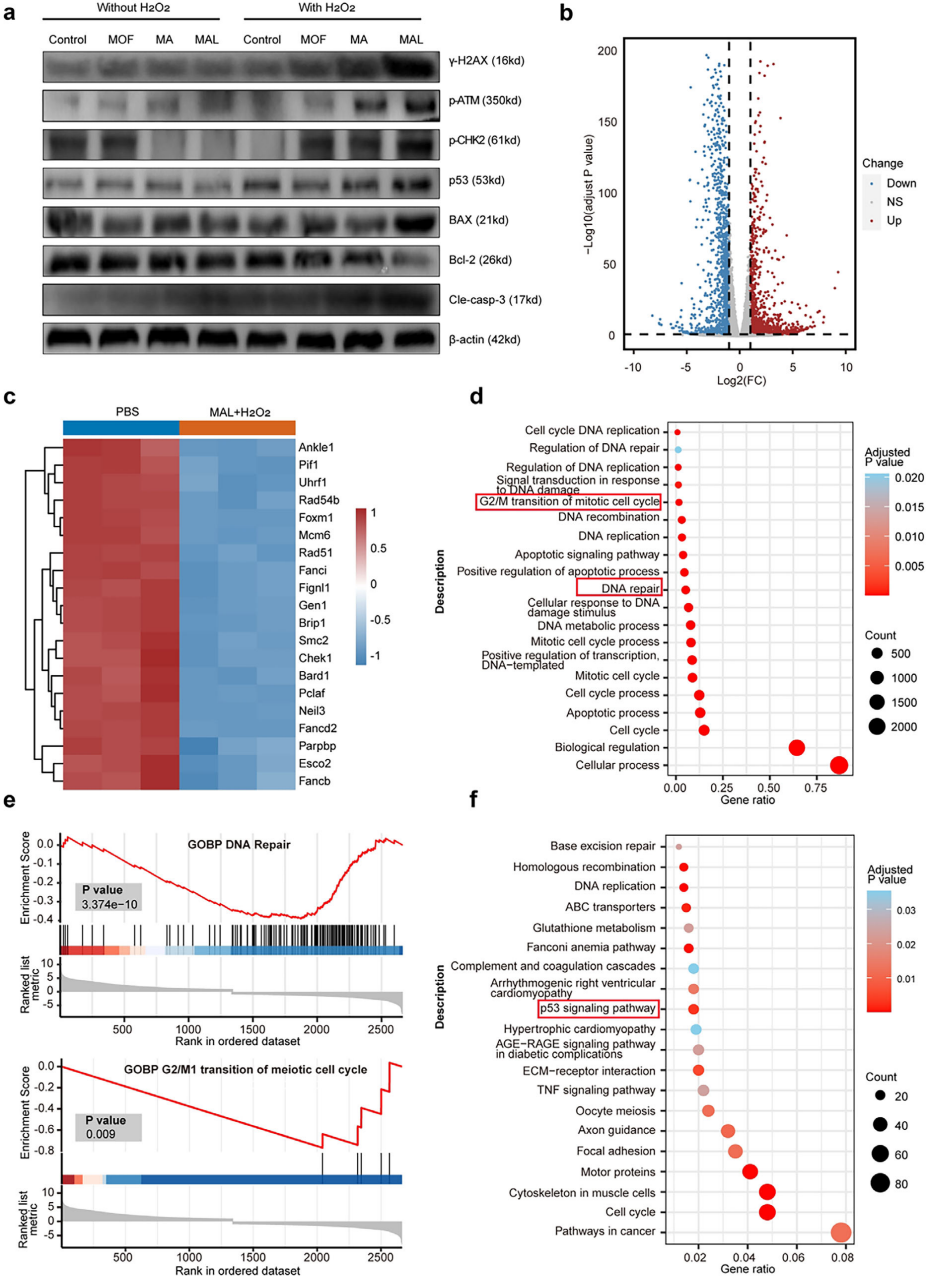
In vitro evaluation of the anticancer activity, mechanism and transcription analysis of MAL. a) WB of CT26 cells following various formulation treatments. b) The genes that were differently expressed between MAL+H_2_O_2_ and PBS were shown by volcano plots. c) DNA repair related gene expression heat map in cells treated with PBS and MAL+H_2_O_2_. d) GO analysis of genes that are differently expressed in CT26 cells treated with PBS and MAL+H_2_O_2_. e) The gene sets for the mitotic cell cycle's G2/M transition and cytosolic DNA repair were revealed by GSEA analysis. f) KEGG pathway enrichment analysis in CT26 cells treated with MAL+H_2_O_2_ and PBS.

DNA damage is known to activate the ataxia telangiectasia mutation/checkpoint kinase 2/p53 (ATM/CHK2/p53) signaling pathway, ultimately activating the caspase‐3 apoptotic protein [58, 59]. Therefore, after different treatments, we evaluated the activation level of the ATM/CHK2/p53 pathway. The WB results showed that the MAL+H_2_O_2_ group significantly increased the expression of p‐ATM, p‐CHK2, and p53 in tumors, indicating the activation of the ATM/CHK2/p53 pathway in tumors. This activated p53 signaling axis subsequently introduces the mitochondrial‐mediated apoptotic pathway. Specifically, we observed through WB experiments that treatment with MAL+H_2_O_2_ resulted in upregulation of pro apoptotic protein BAX and downregulation of anti apoptotic protein Bcl‐2, which further alter mitochondrial membrane permeability and ultimately activate caspase‐3 apoptotic protein to induce mitochondrial‐mediated apoptosis, and amplifying endogenous apoptosis in tumor cells (Figure 4a; Figure S20).

The significant loss of mitochondrial membrane potential further confirms the downstream execution of apoptosis and confirms the involvement of intrinsic apoptotic pathways. Therefore, the mechanism of p53 activation after MAL treatment has been elucidated to be driven by ATM induced DNA damage mediated by CHK2 and executed through the mitochondrial‐mediated apoptotic pathway. Beyond inducing direct DNA damage, we sought to determine whether ONOO^−^ could also impair the cellular repair response. We focused on poly (ADP‐ribose) polymerase 1 (PARP1), a key sensor detection and repair of DNA [60, 61]. Western blot analysis revealed a significant downregulation of PARP1 protein in cells treated with MAL+H_2_O_2_ (Figure S21). This suppression of a major DNA repair enzyme provides a direct mechanistic insight: ONOO^−^ not only generates DNA lesions but also cripples the cell's ability to repair them. This dual action—simultaneous damage infliction and repair pathway inhibition—potentiates the cytotoxic effect and contributes to the strong anti‐tumor efficacy observed.

In order to explore further anti‐cancer mechanisms of MAL, CT26 cells treated in various ways were subjected to whole genome RNA‐seq analysis. The results of principal component analysis, differential gene volcano map and differential gene clustering heatmap show that, compared with the PBS group, MAL+H_2_O_2_ significantly induced gene changes, with red dots indicating genes that are up‐regulated and blue dots indicating genes that are down‐regulated (Figure 4b; Figure S22a,b). The enrichment analysis of GO (Gene Ontology) showed that MAL+H_2_O_2_ mainly affects DNA repair, cell response to DNA stimulation, cell division, and G2/M transition in the mitotic cell cycle (Figure 4d). To learn more about how MAL+H_2_O_2_ affects DNA repair and the cell cycle, gene enrichment analysis (GSEA) and DNA repair related gene heatmap analysis were conducted. The results showed that MAL+H_2_O_2_ could significantly downregulate genes related to DNA repair, G2/M transition in the mitotic cell cycle (Figure 4c,e), and inhibit DNA division (Figure S22c). The KEGG (Kyoto Encyclopedia of Genes and Genomes) enrichment data, MAL+H_2_O_2_ influences DNA replication and p53 signaling pathway gene expression (Figure 4f). In summary, MAL+H_2_O_2_ exerts its anti‐cancer effect by inducing cell cycle arrest, inhibiting DNA repair and affecting the ATM/CHK2/p53 pathway, which is attributed to the cascade production of ONOO**^−^** [62].

### 2.5 Antitumor Effect In Vivo

**FIGURE 5:**
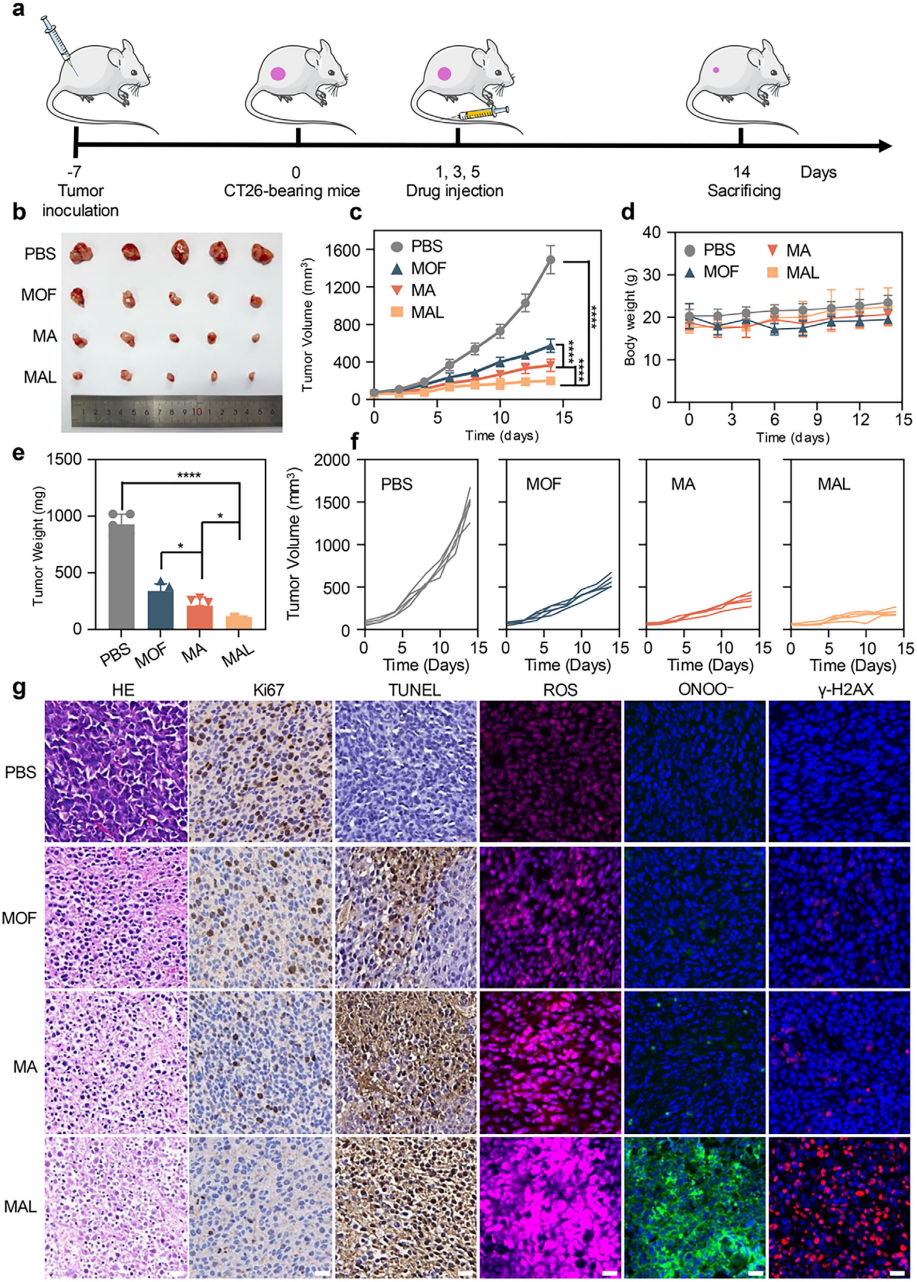
Antitumor activity of MAL in vivo. a) Diagram of CT26 tumor‐bearing mice model. b) Picture of the dissected tumor after treatment. c) Tumor volume curves. d) Body weight trends of mice (n = 5, mean ± SD). e) Weight of dissected tumors. f) Growth curves of BALB/c mice with CT26 tumors in various treatment groups. g) Examples of tumors from the treated mice stained with TUNEL, Ki67, and H&E. Images of tumor tissues stained with ROS, ONOO**^−^**, and γ‐H2AX following two days of various therapies. Scale bar: 20 µm. Data were shown as mean ± SD (n = 3). ^*^ *p* < 0.05, ^**^ *p* < 0.01, ^***^ *p* < 0.001, ^****^ *p* < 0.0001.

To validate the tumor‐targeting capability and biodistribution of MAL, we conducted an in vivo imaging study using Cy5.5‐labeled MAL. Fluorescence imaging revealed rapid accumulation of MAL at the tumor site within 4 h post‐injection, attributable to the EPR effect prevalent in solid tumors [17]. While the signal intensity within the tumor moderated by 8 h, a detectable signal persisted for up to 72 h, demonstrating prolonged retention critical for therapeutic efficacy. Concurrently, the strong initial signal in the liver, which substantially weakened over 72 h, indicates that hepatic clearance is the primary metabolic pathway for MAL, further corroborating its biocompatibility and clearance potential (Figure S23). Next, we evaluated the efficacy of MAL in vivo. After subcutaneous injection of CT26 cells into mice, different drug treatments were administered for 14 days when the tumor volume was approximately 50–100 mm^3^ (Figure 5a). All mice were randomly divided into 4 groups (n = 5). During the treatment period, tumor volume and mouse weight were recorded every two days, followed by euthanization. After treatment, tumor tissue was collected from different groups for photography and weighing. During the treatment process, in order to assess how NPs affected the development of mice during the therapy, we also monitored the mice in various treatment groups for two weeks and noted changes in tumor volume and body weight. Among them, MAL used for cascading nano‐catalysis and regulating endogenous apoptosis demonstrated the most effective tumor suppressive ability (Figure 5b,e). Statistical analysis of the final tumor volumes revealed that the MAL+H_2_O_2_ group exhibited the most potent inhibitory effect, with a statistically significant reduction (*p* < 0.0001) compared to both the PBS control and the MOF‐only group. The MA+H_2_O_2_ group showed a significant growth suppression compared to the control (*p* < 0.0001), outperforming the MOF+H_2_O_2_ group (Figure 5c,f). Notably, over the 14‐day treatment period, no appreciable changes in body weight were noted, suggesting the minimal negative effects of MAL (Figure 5d).

Subsequently, immunohistochemistry (H&E, Ki67, TUNEL) and immunofluorescence staining (γ‐H2AX, ROS, NO, and ONOO**^−^**) were performed on tumor tissues (Figure 5g; Figures S24 and S25), and the findings demonstrated that MAL can considerably increase the production of ROS, NO, and ONOO**^−^**, thereby causing DNA damage, inhibits cell proliferation and promotes tumor cell apoptosis. Next, WB analysis was also conducted on tumor tissues. The production of ONOO**^−^** can induce DNA damage, and in response to severe or irreparable DNA damage. Additionally, pro‐apoptotic protein production can be triggered by p53 activation. The findings demonstrated that the MAL+H_2_O_2_ group had significantly higher levels of γ‐H2AX expression than the other intervention groups. Subsequently, the p53 pathway was then activated, leading to a large upregulation of the pro‐apoptotic protein BAX and a significant downregulation of the anti‐apoptotic gene Bcl‐2 (Figure S26). These results demonstrate that MAL can activate the p53 pathway through the production of ONOO**^−^**, thereby encouraging tumor cell death and enhancing the tumors' therapeutic efficacy. In addition, H&E staining of mouse organs, liver function indicators, and blood routine results showed that MAL did not cause significant damage or inflammatory lesions (Figures S27–S29). To evaluate the potential long‐term toxicity of MAL, a 30‐day biosafety study was performed in healthy mice using the same administration route and dose as the treatment model. Body weight was monitored regularly, and at the end of the study, histopathological analysis of major organs and comprehensive hematological and serum biochemical tests were conducted. The results revealed no significant weight loss, no pathological abnormalities in organ tissues, and no notable deviations in blood parameters (Figures S30–S33). These findings indicate that MAL possesses favorable long‐term biocompatibility and does not elicit cumulative toxicity in vivo, supporting its potential for safe therapeutic application. These results indicate that MAL has good biocompatibility and anti‐tumor effects.

## 3 Conclusion

In this work, we have developed a new cascade nanocatalytic approach using the designed nanomedicine MOF‐Au‐L‐Arg (MAL) to improve cancer treatment. By integrating gold nanoparticles into MOF and loading it with L‐arginine, a NO donor, MAL significantly amplifies the catalytic production of ROS. These ROS, through a cascade reaction, interact with NO to form ONOO**^−^**, a highly cytotoxic species with superior tumor cell toxicity and a longer diffusion range compared to ROS. Importantly, ONOO**^−^** synthesized in situ by tumor cells exhibits higher cytotoxicity and can activate the ATM/CHK2/p53 signaling pathway by causing DNA damage. The expression of the anti‐apoptotic protein Bcl‐2 is inhibited and the apoptotic protein BAX is further promoted by p53 pathway activation. The activation of these proteins further induces mitochondrial dysfunction and ultimately triggers mitochondrial‐mediated apoptosis through caspase‐3 apoptotic protein, expanding endogenous apoptosis in tumors. ONOO**^−^** can also inhibit DNA repair by PARP1 and interfere with the cell cycle to promote tumor cell apoptosis. The therapeutic effect of CDT is greatly increased by the ONOO**^−^** generated by the intratumoral cascade working in concert with the ROS storm. Experiments conducted in vitro and in animals have shown that MAL efficiently slows the growth of tumors without causing serious systemic side effects. This work provides a new paradigm for enhancing nanocatalytic therapy through cascade reactions, facilitating durable, DNA‐focused cytotoxicity, and promoting effective programmed apoptosis. The successful integration of cascade nanocatalysis with the p53 pathway activation holds immense potential for improving the outcomes of CDT cancer therapies, particularly those based on catalytic drug delivery systems, and it makes it possible to create more effective and targeted cancer treatments. Prospectively, we will explore the potent antitumor effects of ONOO^−^, particularly its role in driving immunogenic cell death and reprogramming the tumor immune landscape.

## Conflicts of Interest

The authors declare no conflicts of interest.

## Ethics Statement

We promise that the study was performed according to the international, national and institutional rules considering animal experiments. The study protocol of animal was approved by Animal Ethics Review Committee of Tongji University (TJBH08425106).

## Supporting information




**Supporting File**: advs74086‐sup‐0001‐SuppMat.docx.
[Correction added on 06 February 2026, after first online publication: the title of the Supporting Information has been changed.]

## Data Availability

The data that support the findings of this study are available in the supplementary material of this article.
