# Supplementary material for: Endogenous Amplification of Apoptosis via p53 Regulation using a Cascade Nanocatalytic Medicine
Source: Adv Sci (Weinh). 2026 Feb 11;13(19):e20367. doi: 10.1002/advs.202520367 (PMC13045313; doi:10.1002/advs.202520367)
Supplement: Supplementary file 1 — Supporting File: advs74086‐sup‐0001‐SuppMat.docx. [Correction added on 06 February 2026, after first online publication: the title of the Supporting Information has been changed.] [file ADVS-13-e20367-s001.docx]

**Supporting Information**

**Endogenous Amplification of Apoptosis via p53 Regulation using a Cascade Nanocatalytic Medicine**

Tan Wu^1^, Xiaoyue Xu^1^, Dan Xu^3^, Hui Zhang^1^, Nan Wang^1^, Yang Yang^1^, Qian Chen^1,^*, Shunjie Chen^2,^*, Cheng Li^1,^*

^1^Department of Anesthesiology and Perioperative medicine, Shanghai Key Laboratory of Anesthesiology and Brain Functional Modulation, Clinical Research Center for Anesthesiology and Perioperative Medicine, Translational Research Institute of Brain and Brain-Like Intelligence, Shanghai Fourth People's Hospital, School of Medicine, Tongji University, Shanghai 200434, China.

^2^Department of Nephrology, Shanghai Fourth People's Hospital, School of Medicine, Tongji University, Shanghai 200434, China.

^3^Xinhua Hospital Affiliated to Shanghai Jiao Tong University School of Medicine, Shanghai 200092, China

E-mail: [chengli_2017@tongji.edu.cn](mailto:chengli_2017@tongji.edu.cn)

E-mail: chenshunjie77csj@163.com

E-mail: [chenqian1989@tongji.edu.cn](mailto:chenqian1989@tongji.edu.cn)

**Materials**

Ferric (III) chloride hexahydrate, 2-aminoterephthalic acid, Methylene blue, 1-naphthol, L-arginine, 2,3-Butanedione and fluorescein isothiocyanate (FITC) were purchased from Aladdin. N, N-dimethylformamide (DMF) and acetic acid were purchased from Macklin. Sodium borohydride was obtained from Yunnan Jingrui Technology Co., Ltd. Tetrachloroauric acid trihydrate was obtained from Shanghai Yuanye Biotechnology Co., Ltd. Dulbecco's modified Eagle's medium (DMEM), Fetal bovine serum (FBS), penicillin−streptomycin and trypsin-EDTA (0.25%) were provided by Gibco. 4′,6-diamidino-2-phenylindole dihydrochloride (DAPI), Reactive Oxygen Species (ROS) Assay Kit, Griess Reagent, Nitric Oxide Assay Kit with DAF-FM DA, Calcein/PI Viability/Cytotoxicity Assay Kit, Comet Assay Kit, Mitochondrial membrane potential assay kit with JC-1, Hydrogen Peroxide Assay Kit, and Annexin V-FITC Apoptosis Detection Kit were purchased from Beyotime. Peroxynitroso anion (**ONOO^–^**) detection kit was obtained from Bestbio. Anti-gamma H2A.X, Anti-Bcl-2, Bax (D3R2M) Rabbit mAb, p-ATM (Ser1981) (10H11.E12), PARP Antibody, Cleaved Caspase-3 (Asp175) Antibody, and p53 (1C12) Mouse mAb were purchased from Abcam and Cell Signaling Technology. p-CHEK2 (Thr68) Recombinant monoclonal antibody was obtained from Proteintech. All the mice were maintained under specific pathogen free (SPF) conditions after purchasing from Sibeifu (Suzhou) Biotechnology Co., Ltd.

**Synthesis of MOF**

The prior literature was consulted in the synthesis of the structurally optimized MOF NPs. In particular, FeCl_3_·6H_2_O (1.122 g) and 2-aminoterephthalic acid (0.756 g) were combined in 90 mL of DMF while being stirred until they were fully dissolved. Acetic acid (1.188 mL) was then added to the mixture dropwise. The system should then be placed at 120 °C and stirred for four hours. To get rid of extra reactants and contaminants, wash once with DMF and again with anhydrous ethanol at 13,000 rpm.

**Synthesis of MA**

Add a 2:1 mass radio HAuCl_4_·3H_2_O solution to the MOF and stir at 600 rpm for half an hour. Subsequently, sodium borohydride was added to ultrapure water that had been pre cooled at 4 °C. After complete dissolution, mix and dissolve it, and continue stirring for half an hour. After centrifugation, wash with ultrapure water three times to obtain MA.

**Synthesis of MAL**

Add L-Arg dissolved in ultrapure water to MA under a stirring ring. After stirring for 24 hours under dark conditions, centrifuge and wash three times with ultrapure water to obtain MA. Use the naphthol diethylhydrazine method to determine the amount of L-Arg present in the supernatant. To put it briefly, the indicator aqueous solution is made with NaOH (40 mg mL^-1^), 1-naphthol-propanol (80 mg mL^-1^), and diacetyl propanol (0.5 μL mL^-1^). 20 μL of supernatant and 130 μL of indicator solution were mixed, then the absorbance of the above mixture was detected at 540 nm after 20 minutes incubation.

**Characterization**

Take TEM pictures and EDS elemental spectra with a Tecnai G2 F30 electron microscope. A Zetasizer Nanoseries (Malvern, UK) was used to measure the DLS size and ZETA potential. K-Alpha (Thermal Scientific) was used for XPS. The UV-2700i (Shimadzu, Japan) was used to record the nanoparticles' UV-vis spectra. IR Spirit Shimadzu of Japan acquired the FTIR spectrum. Inductively coupled plasma optical emission spectroscopy (ICP-OES, Agilent Technologies) is used to measure the concentration of iron ions in MOF, MA, and MAL nanoparticles.

**Determination of Extracellular ROS generation**

Using methylene blue to evaluate the ability of nanoparticles to generate ROS. Incubate different concentrations of MOF, MA, and MAL (20, 50, 100, 200, and 400 μg mL^-1^) with MB (10 μg mL^-1^) for 10 minutes with or without H_2_O_2_ (100 μM). Subsequently, a UV spectrometer was used to measure the UV spectral changes of methylene blue at wavelengths of 500-800 nm. Next, DMPO is used to detect •OH and O_2_^•−^. DMPO (40 mM) is usually added to a MES buffer solution that contains MOF, MA, and MAL. H_2_O_2_ is then added or left out. Further track the •OH and O_2_^•−^ signal using an electron paramagnetic resonance spectrometer.

**Detecmination of the catalytic efficiency of nanoparticles**

Using methylene blue to evaluate the ability of nanoparticles. Incubate different concentrations of MOF, MA, and MAL (50 μg mL^-1^) with MB (10 μg mL^-1^) for 5 minutes with H_2_O_2_ (0.1, 0.2, 0.5, 1, 2, and 5 mM). Subsequently, a UV spectrometer was used to measure the UV spectral changes of methylene blue at wavelengths of 500-800 nm.

**DFT calculations**

DFT calculations were performed using the Vienna ab initio simulation package (VASP). The generalized gradient approximation (GGA) in the form of Perdew-Burke-Ernzerhof (PBE) was adopted as the exchange correlation functional. The energy cutoff of 450 eV and the k-point meshes of 1×1×1 were proposed to carry out geometry optimization and electronic structure calculation. During the geometry optimization, the entire system is considered to have successfully converged until the convergence thresholds of maximum force and energy were smaller than 0.05 eV/ A and 1.0×10^-5^ eV/atom, respectively. The vacuum slab was set as 10Å to avoid interactions between neighboring structures. Spin-polarisation calculation is for the calculation of electronic structures.

**Extracellular NO generation**

To detect the production of NO, different concentrations of MAL (100, 200, 300 μg mL^-1^) were mixed with or without the addition of H_2_O_2_, and then detected using a nitric oxide kit (Beyotime). After two hours of reaction, centrifuge and collect the supernatant and measure the absorbance at 540 nm with a SpectraMax M5 microplate reader.

**Determination of Extracellular ONOO**^–^ **generation**

Using the **ONOO^–^** assay kit (BestBio) to detect the production of **ONOO^–^** in vitro. Dilute the fluorescent probe by 10 times and add 10 μL of the probe to each group. Incubate at 37 °C for 2 hours and use a fluorescence spectrometer to detect. The maximum excitation wavelength is 516 nm.

**Cell Culture**

Mouse colorectal cancer cells (CT26) were cultured in DMEM medium supplemented with 10% FBS and 1% streptomycin/Penicillin at 37 °C and 5% CO_2_ conditions.

**In vitro cytotoxicity assay**

After being seeded into a 96-well plate, CT26 cells were incubated for 24 hours with varying doses of MOF, MA, ML, and MAL (0, 5, 12.5, 25, 50, 100, and 200 μg mL^-1^). For a while, keep incubation with DMEM containing 10% CCK-8 test without serum. Next, use a SpectraMax M3 microplate reader to measure the absorbance at 450 nm.

After being seeded into a 96-well plate, CT26 cells were exposed to different formulations of MOF, MA, ML, and MAL for 24 hours (0, 5, 12.5, 25, 50, 100, and 200 μg mL^-1^). Following a period of incubation with DMEM containing 10% CCK-8 test, use a SpectraMax M3 microplate reader to detect the absorbance at 450 nm.

**Determination of Intracellular ROS generation**

CT26 cells were seeded into a 12 well plate. After a 24 h incubation. The cells were treated with 50 μg mL^-1^ concentration of various formulations (PBS, MOF, MA, and MAL) with or without H_2_O_2_ for 24 h. CLSM and flow cytometry were used to detect intracellular ROS levels after all cells had been treated with DCFH-DA (Beyotime) for 20 minutes at 37 °C in the dark.

**Mitochondrial membrane potential detection**

CT26 cells were seeded into a 12 well plate. After a 24 h incubation. The cells were treated with 50 μg mL^-1^ concentration of various formulations (PBS, MOF, MA, and MAL) with or without H_2_O_2_ for 24 h. JC-1 was added to each group and incubated at 37 °C in the dark for 20 minutes. It was washed three times with staining buffer solution and then observed using CLSM.

**Determination of Intracellular NO and ONOO^–^** **generation and DNA damage**

NO probe (Beyotime) and **ONOO^–^** probe (BestBio) were used to detect the intracellular NO and **ONOO^–^**. Briefly, CT26 cells were seeded in a 12 well plate containing slides for 24 h. The cells were treated with 50 μg mL^-1^ concentration of various formulations (PBS, MOF, MA, and MAL) with or without H_2_O_2_ for another 24 h. All cells were treated with **ONOO^–^** probe for 30 min and then incubated with DAPI for 10 min. Subsequently, the levels of intracellular NO and **ONOO^–^** were observed by CLSM and flow cytometry. For detection of DNA damage, the cells after nanoparticel incubation were labeled with anti-γ-H2AX antibody, and the nucleus was stained with DAPI, followed by imaging through confocal microscopy.

**Live/dead cell staining assay and Cellular Apoptosis Detection**

CT26 cells were seeded in a 12 well plate containing slides. After that the cells were treated with various formulations (PBS, MOF, MA, and MAL) (50 μg mL^-1^) with or without H_2_O_2_. The cells were then treated for 30 minutes with the Annexin V-FITC/PI Apoptosis Detection Kit (Beyotime) and Calcein-AM/PI (Beyotime). After that, CLSM and flow cytometry were used to analyze the cell death.

**Western Blot in vitro**

CT26 cells were seeded in 6-well plates. Different nanoparticles were added into the wells. Following a 24-hour incubation period, the cells underwent three rounds of cold PBS washing. The cell solution was then mixed with the RIPA lysis buffer that contained protease and phosphatase inhibitors. Cell proteins were isolated using a centrifuge set to 12,000 rpm for 10 minutes. The BCA (Beyotime) protein assay kit was used to quantify the protein concentration. The proteins were then moved to PVDF films and treated with the antibodies against different proteins for a whole night at 4 °C on a shaker. The electrophoresis procedure was then carried out using SDS-PAGE using a gel-electrophoretic device. Following three rounds of washing, the PVDF films were incubated for one hour with HRP labeled antibodies. UVP ChemStudio (USA) used the ECL chemiluminescent reagent (Beyotime) to create the Western blot images.

**RNA-seq analysis**

CT26 cells were seeded in 6-well plates. Then the cells were treated with different treatments in the culture medium at 37 °C for 24 h, respectively. Agilent 2100 Bioanalyzer was used for RNA-seq. When p-values were less than 0.05 and fold changes were larger than or equal to 1, DEGs were detected. Ggplot2 and heatmap were employed to generate volcano graphs and the heatmap, respectively. With Phyper, the KEGG enrichment analysis was finished. Significant enrichments were identified when the p-values were less than or equal to 0.05.

**In Vivo Fluorescence Imaging**

After each mouse had an intravenous injection of MAL, the mice were put to sleep at the appropriate intervals (0, 4, 8, 24, 48, and 32 hours), and the tumors and main organs were removed for fluorescence imaging using VISQUE InVivo Elite (Vieworks, Republic of Korea).

**In Vivo Antitumor Study**

Four groups of Balb/c mice with CT26 tumor cells were randomly assigned. (n = 5): PBS, MOF, MA, and MAL. When the tumor size was between 50 and 100 mm^3^. Every two days, the body weights and tumor sizes of mice were recorded. The tumor volumes were calculated using the following formula: 1/2 × (tumor length) × (tumor width)^2^. On the 14th day, the tumor was removed. Tumor tissue has been preserved in paraformaldehyde and -80 °C for subsequent staining analysis.

**Western blotting**

Tumors were gathered on the third day, and ripa containing phosphatase and protease inhibitors was applied. Following tissue grinding with a tissue grinder, the recovered protein was centrifuged. The SDS-polyacrylamide gel was used to separate the proteins.

**Statistical Analysis**

All experimental data were presented as mean ± standard deviation (SD). One-way analysis of variance (ANOVA) and Two-way analysis of variance were implemented for analysis of statistical significance. The statistical significance was indicated by * for p < 0.05, ** for p < 0.01, *** for p < 0.001, and **** for P < 0.0001. Data were analyzed by using Graphpad.


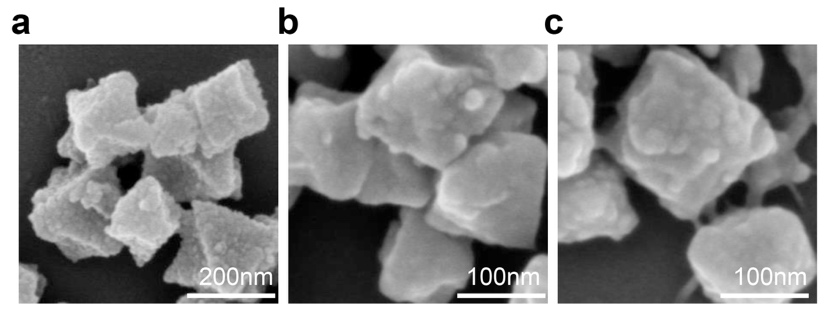


Figure S1: SEM image of a) MOF, b) MA, and c) MAL.


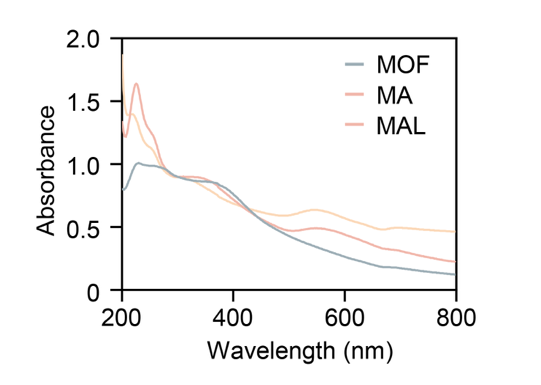


Figure S2: UV–vis absorbance spectra of MOF, MA, and MAL.


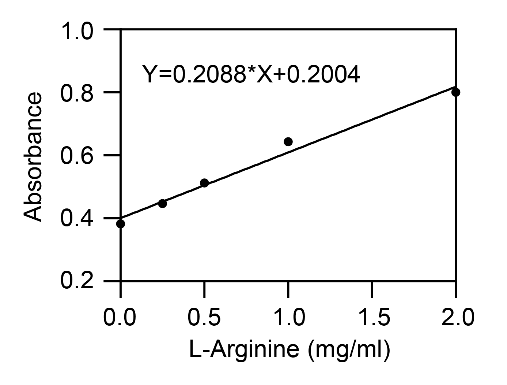


Figure S3: Standard curve of L-Arg.


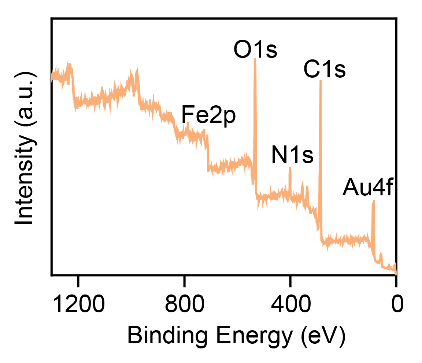


Figure S4: Full-survey XPS spectrum of MAL.


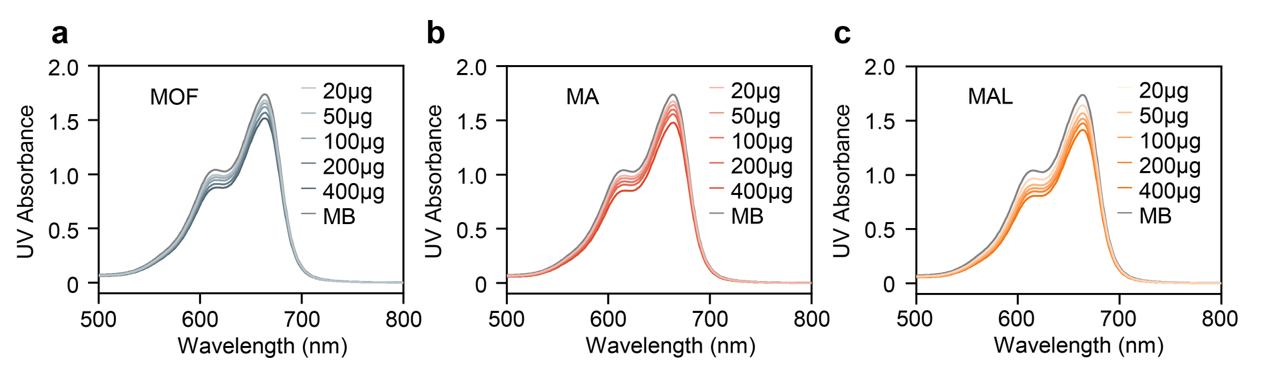


Figure S5: UV–vis absorption spectra of MB after reaction with different concentrations of a) MOF, b) MA, and c) MAL.


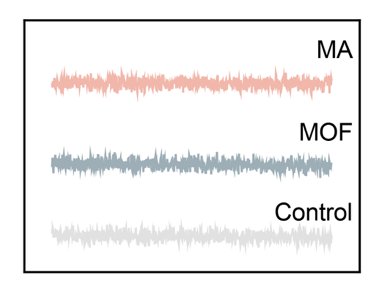


Figure S6: ESR spectra of control, MOF, and MA in the presence of 5,5-dimethyl-1-pyrroline-N-oxide (DMPO) as the spin trap to detect •OH radicals.


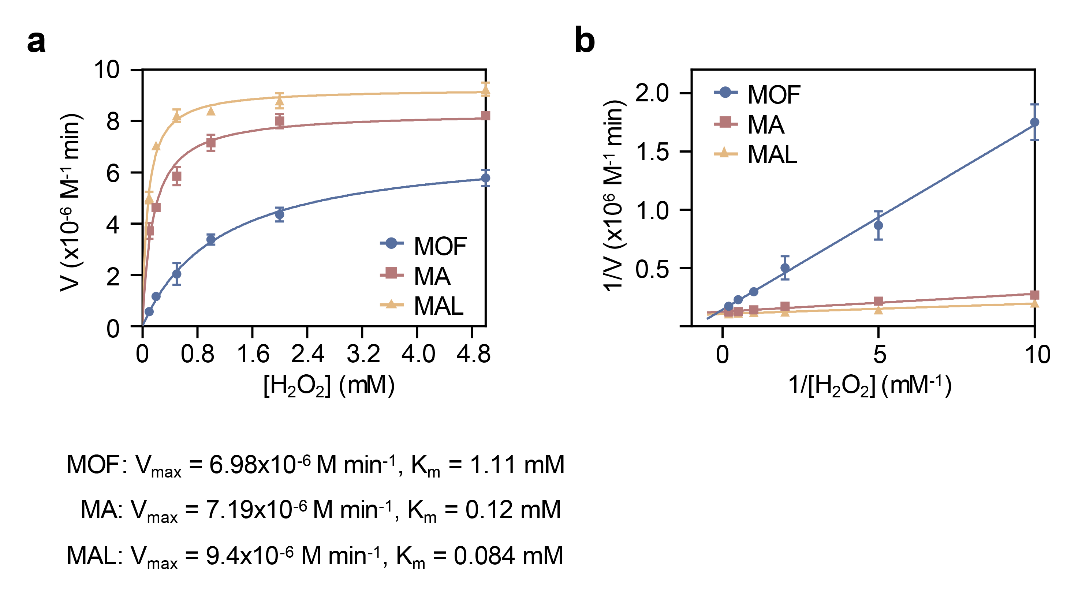


Figure S7: a) Michaelis Menten curve and b) Lineweaver Burk plot of MOF, MA, and MAL.


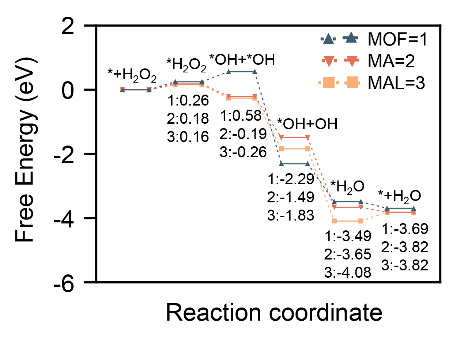


Figure S8: Free energy level diagrams of the Fenton reaction.


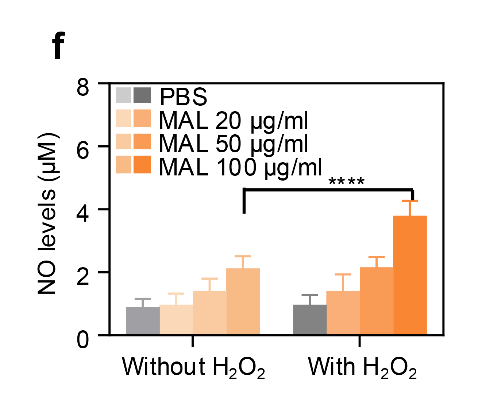


Figure S9: In vitro production of NO.


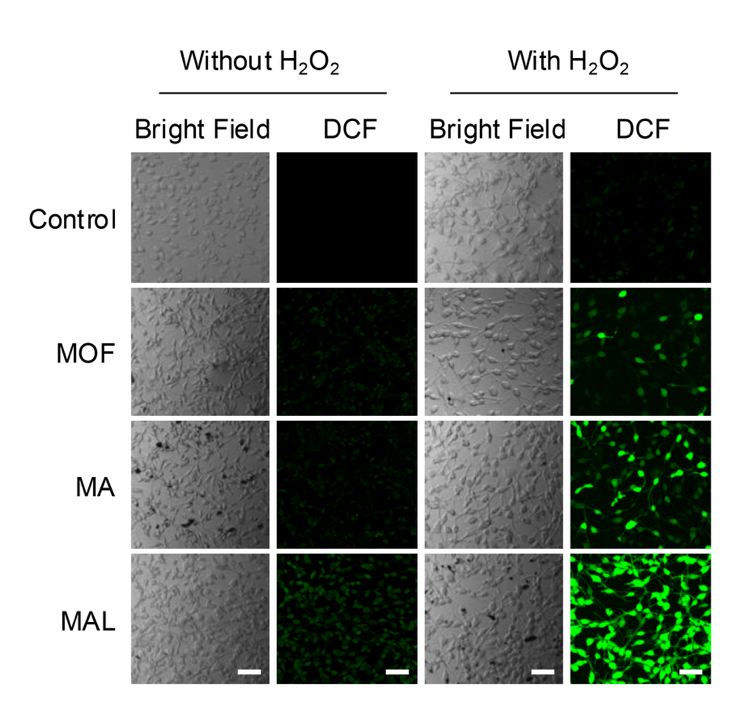


Figure S10: CLSM images showing ROS generation in CT26 cells after different treatments. ROS were detected by DCF (green). Scale bar: 50 μm.


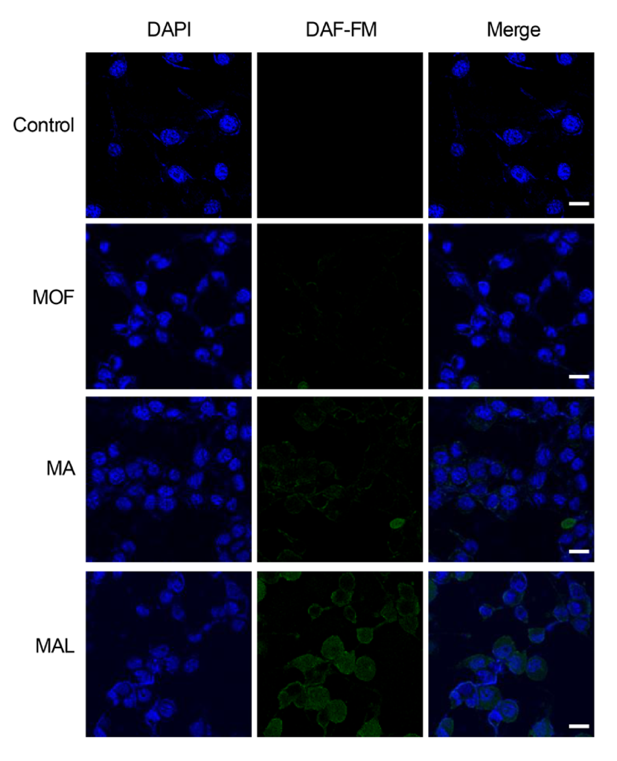


Figure S11: CLSM images of NO staining after different treatments. The nuclei were visualized with DAPI (blue). Scale bar: 20 μm.


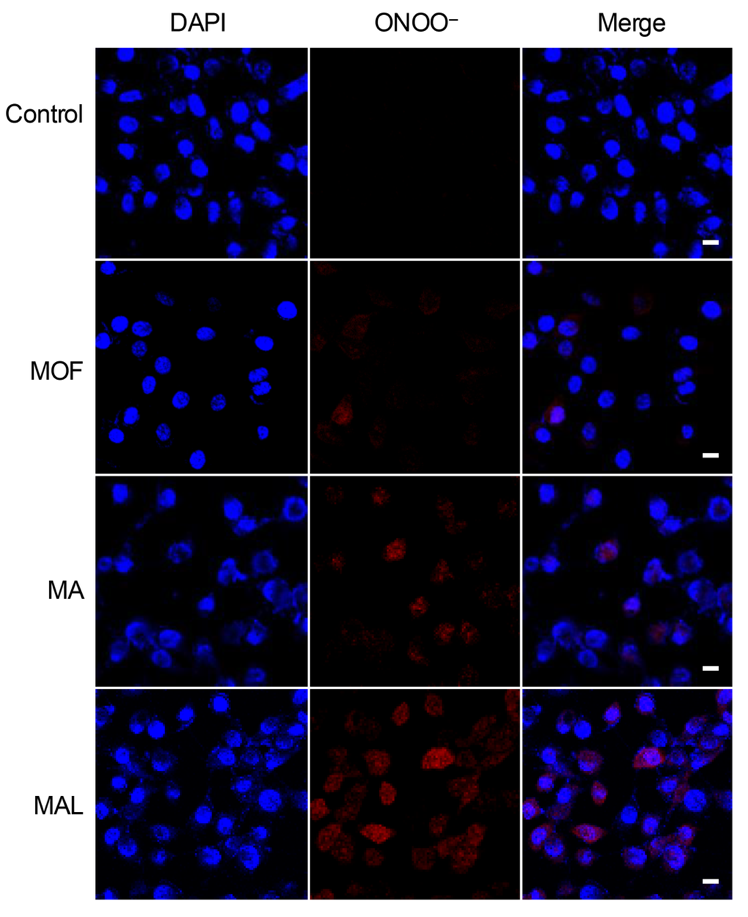


Figure S12: Generation of **ONOO^–^** in CT26 cells as detected by **ONOO^–^** probe, respectively. Scale bar: 20 μm.


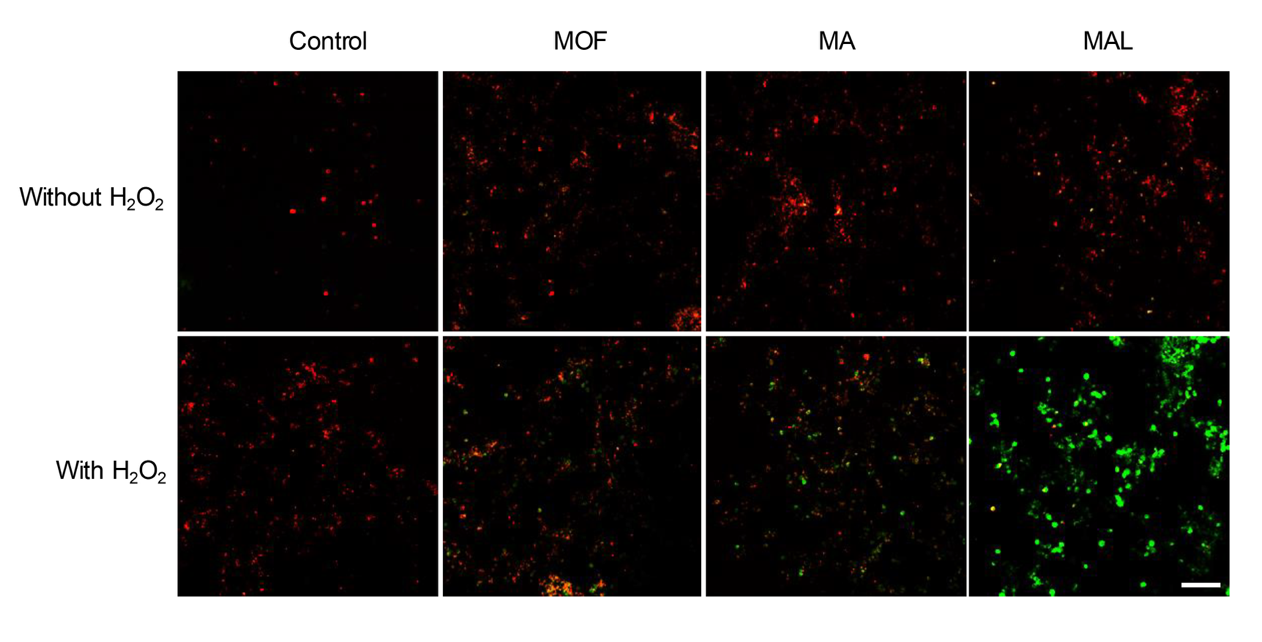


Figure S13: CLSM images of CT26 cells stained with JC-1 after various treatments to detect the changes of mitochondrial membrane potential. Scale bar: 100 μm.


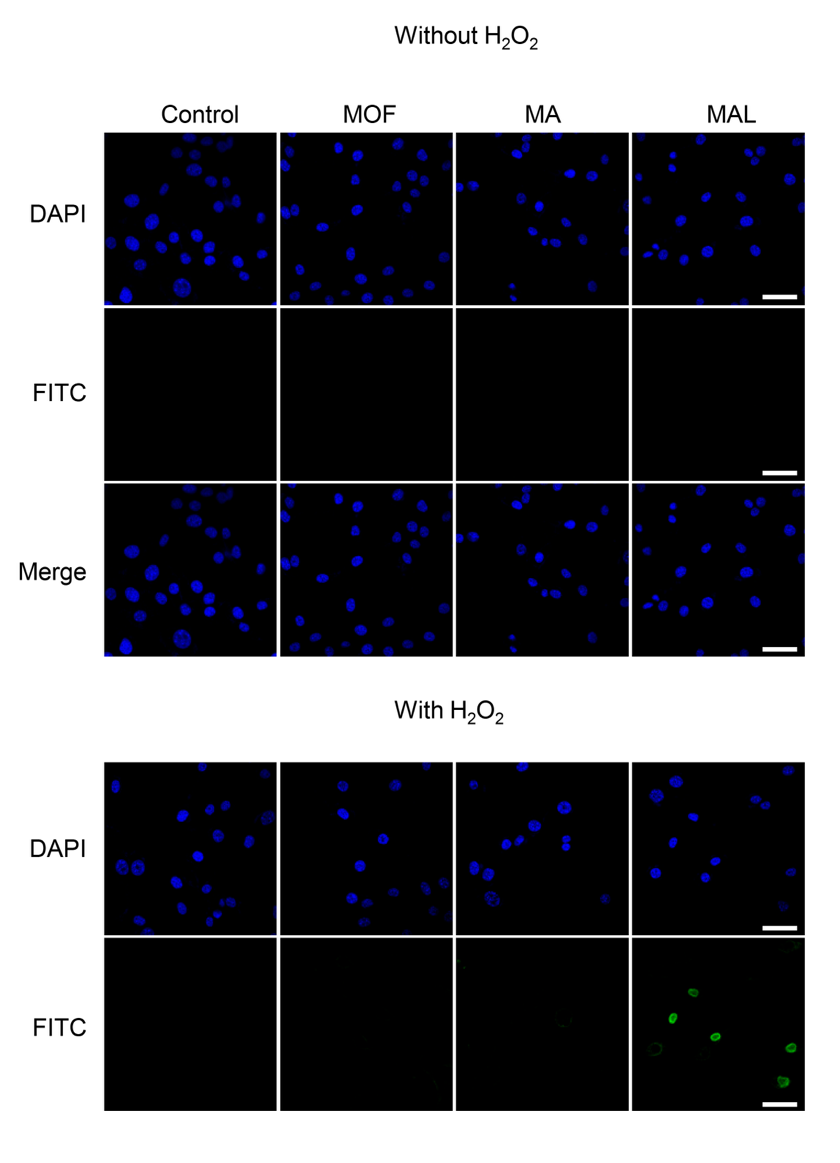


Figure S14: γ-H2AX foci of CT26 cells were detected by γ-H2AX immunofluorescence after treatment. Cell nuclei were stained with DAPI. Scale bar: 20 μm.


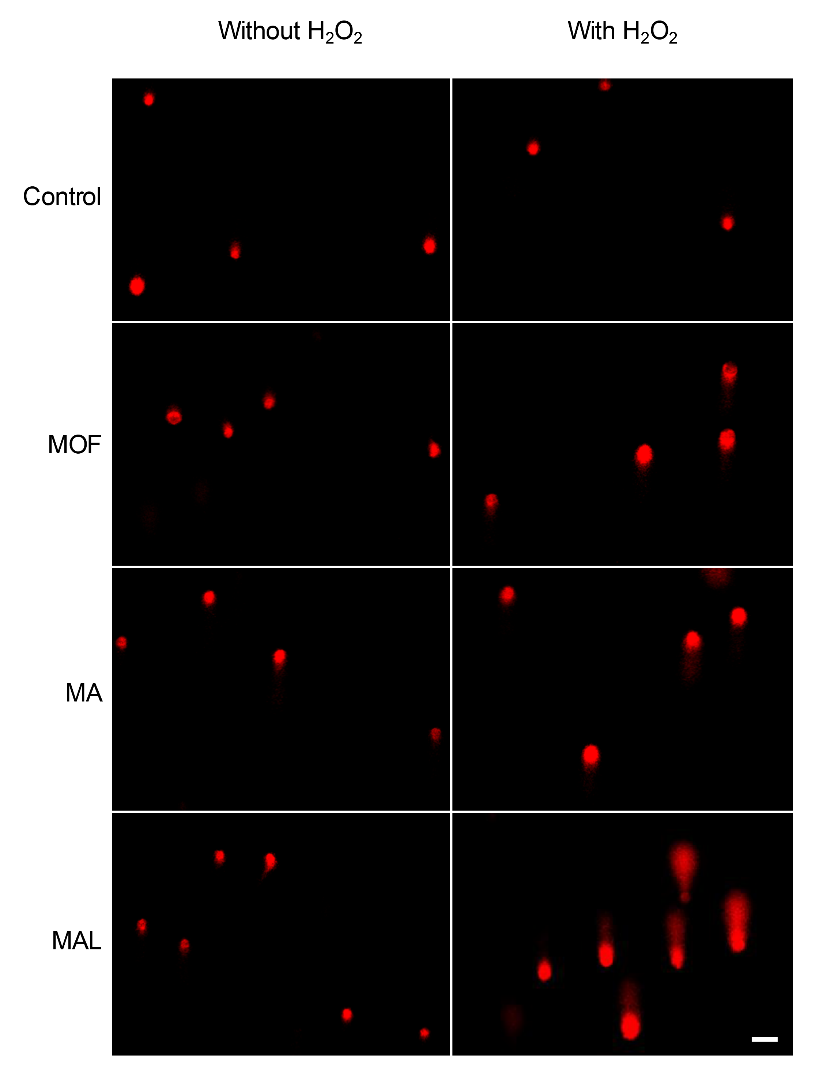


Figure S15: Comet electrophoresis experiments to detect DNA damage. Scale bar: 50 μm.


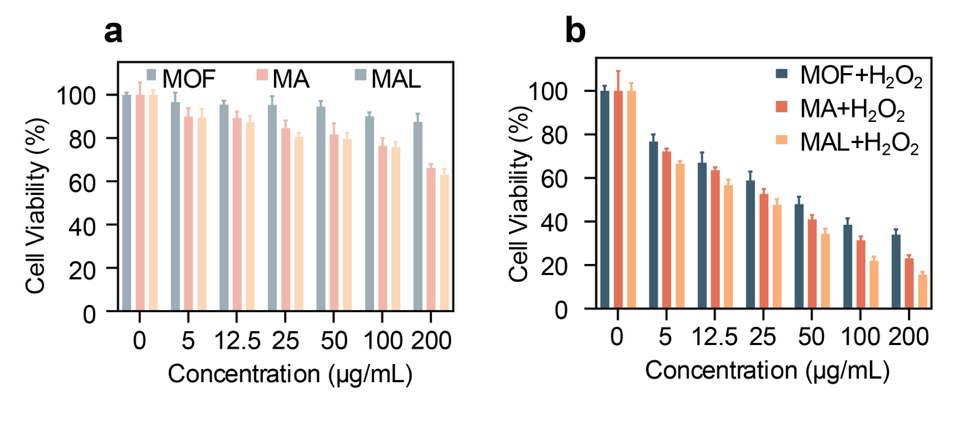


Figure S16: Viability of CT26 cells after treatment with MOF, MA, and MAL in the a) presence, and b) absence of H_2_O_2_ for 24 h.


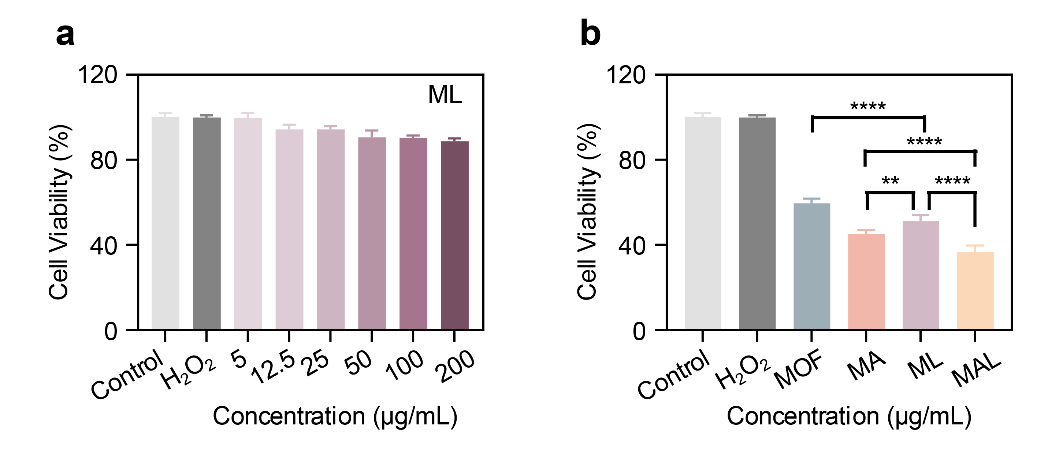


Figure S17: Viability of CT26 cells after treatment with MOF, MA, ML, and MAL in the a) presence, and b) absence of H_2_O_2_ for 24 h.


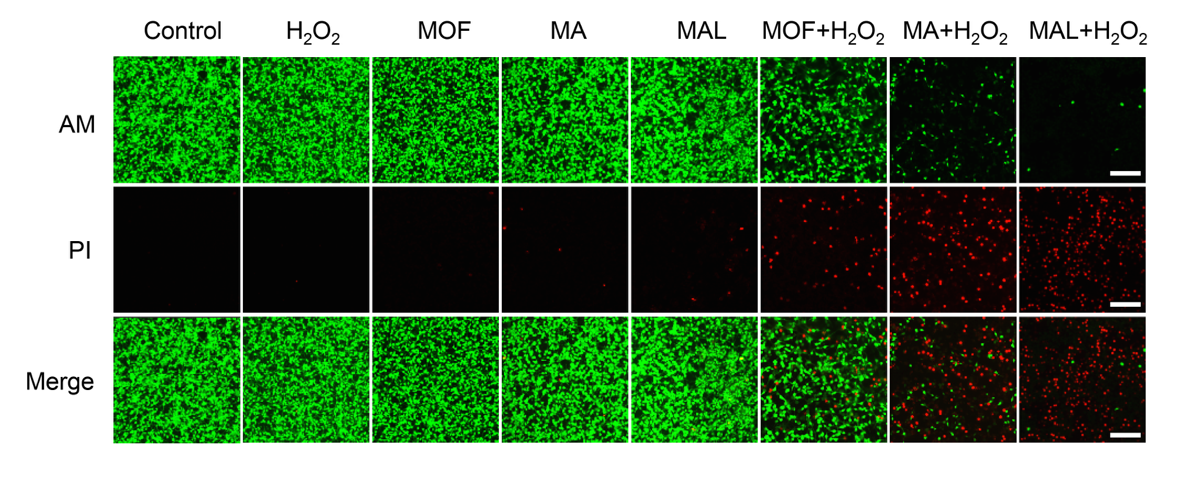


Figure S18: Live/death staining of CT26 cells after different treatments (Green channel: Calcein-AM, Red channel: PI, scale bar: 100 μm, n = 3 biologically independent samples with similar results).


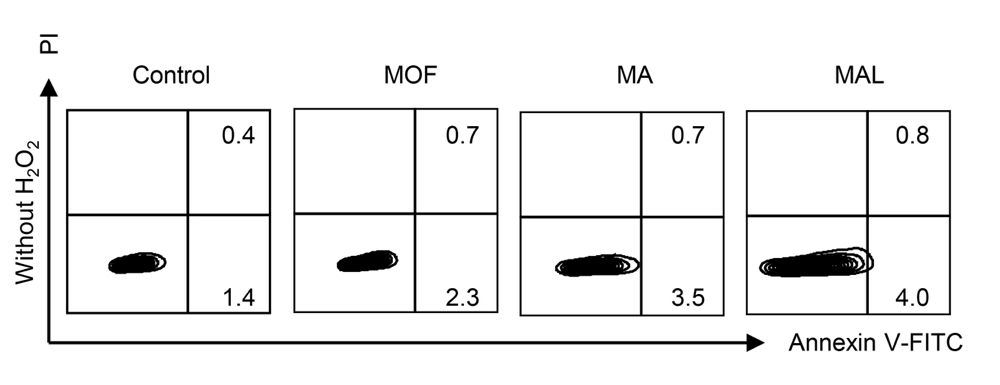


Figure S19: Flow cytometry apoptosis assay of CT26 cells after different treatments.


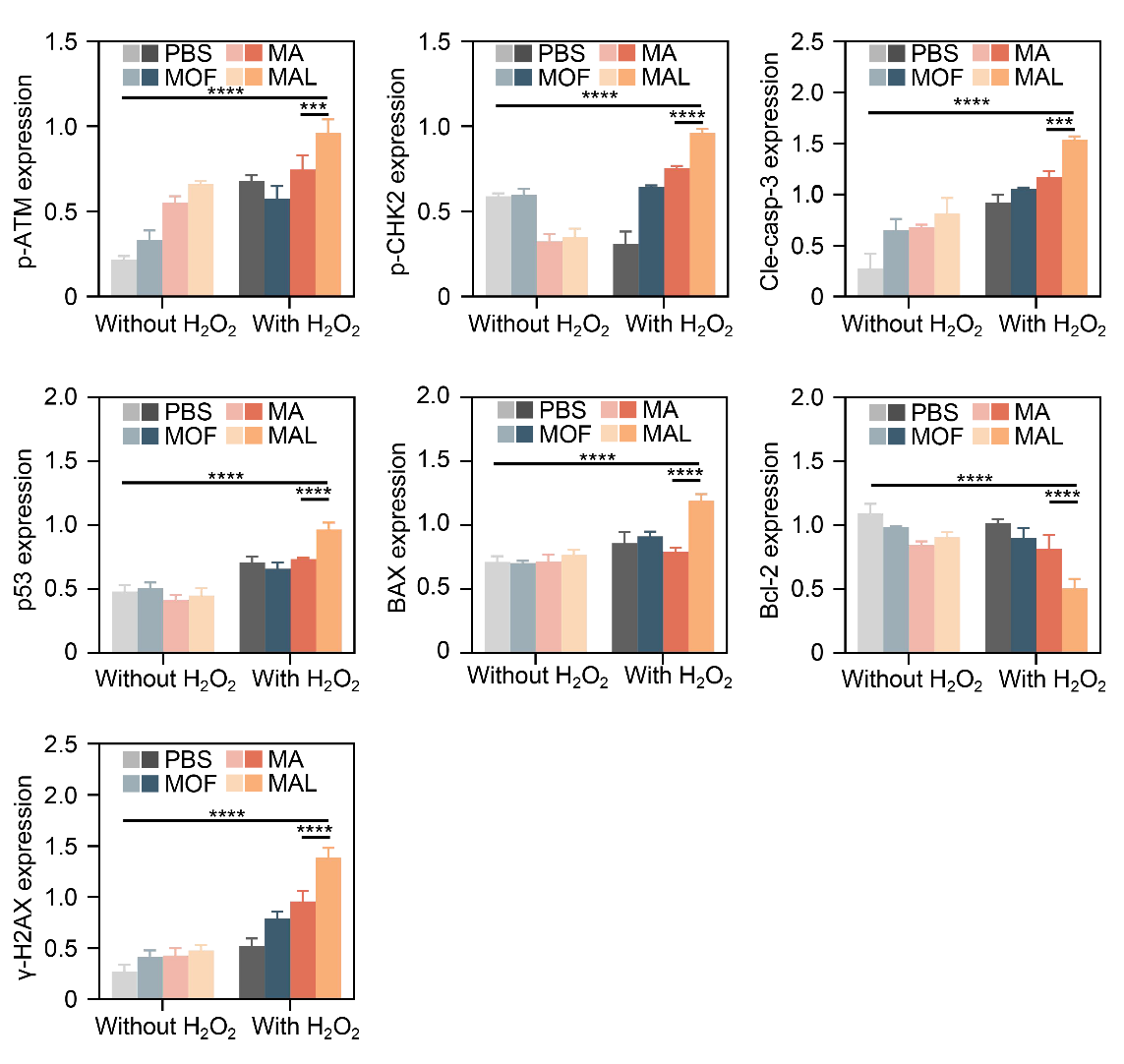


Figure S20: WB analysis of the expression levels of γ-H2AX, p-ATM, p-CHK2, p53, BAX, Bcl-2, and Cle-caspase-3 in CT26 cells with different treatment.


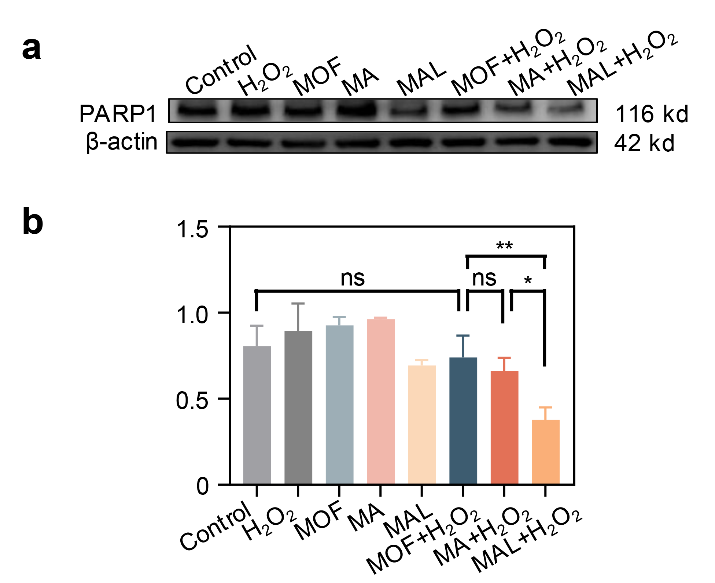


Figure S21: a) WB analysis of the expression of PARP1. b) Quantitative analysis of of PARP1 in (a).


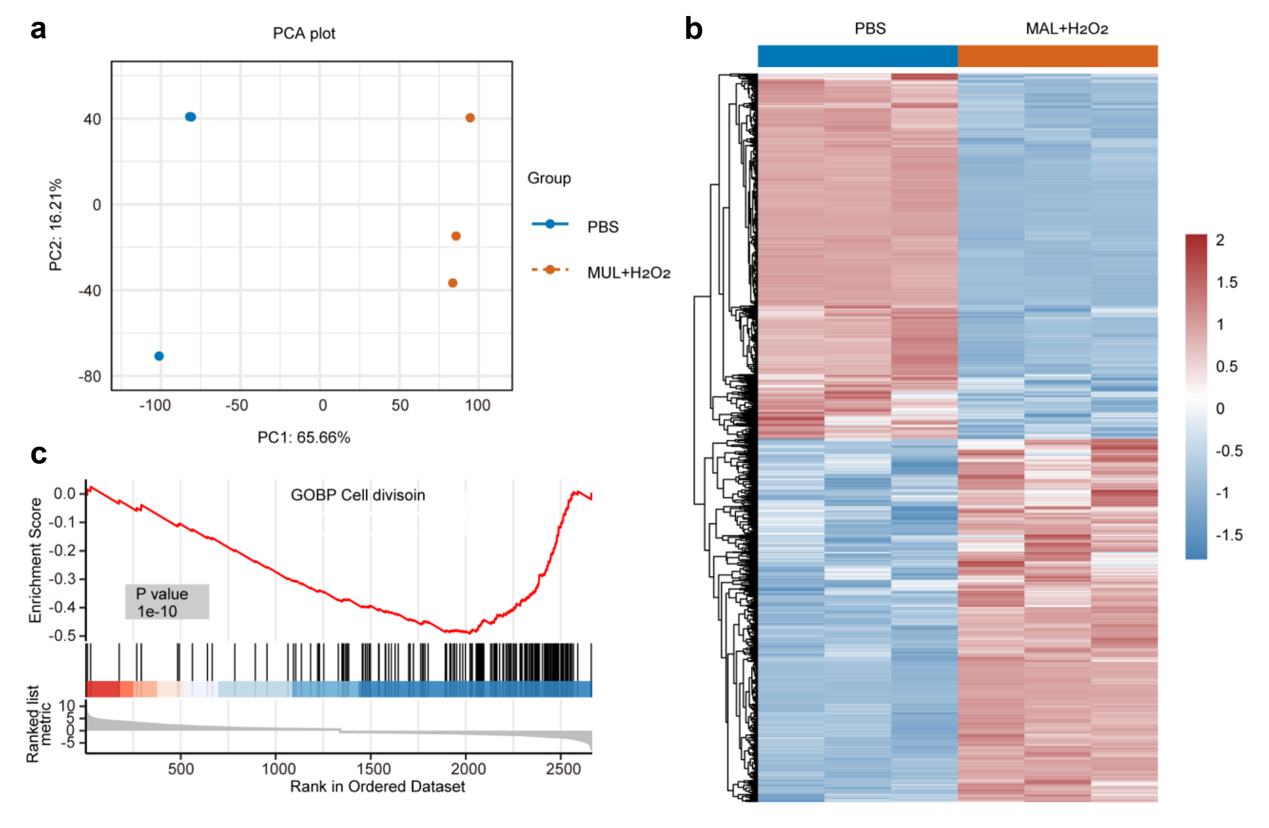


Figure S22: RNA-seq analysis of CT26 cells treated by MAL+H_2_O_2_ and PBS. a) principal component analysis. b) differential gene clustering heatmap. c) gene enrichment analysis (GSEA) of DNA division


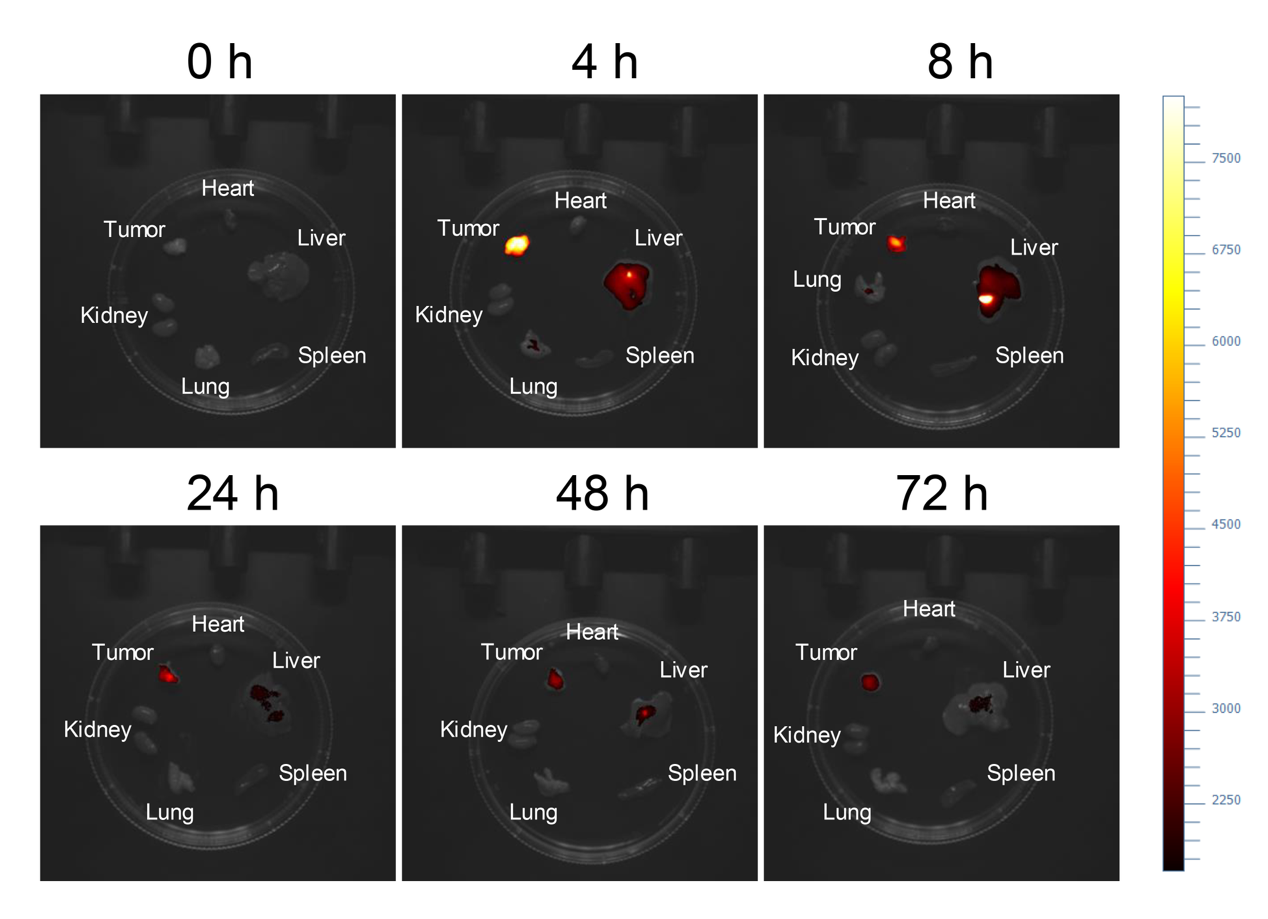


Figure S23: Organ ex vivo imaging at different time points of MAL.


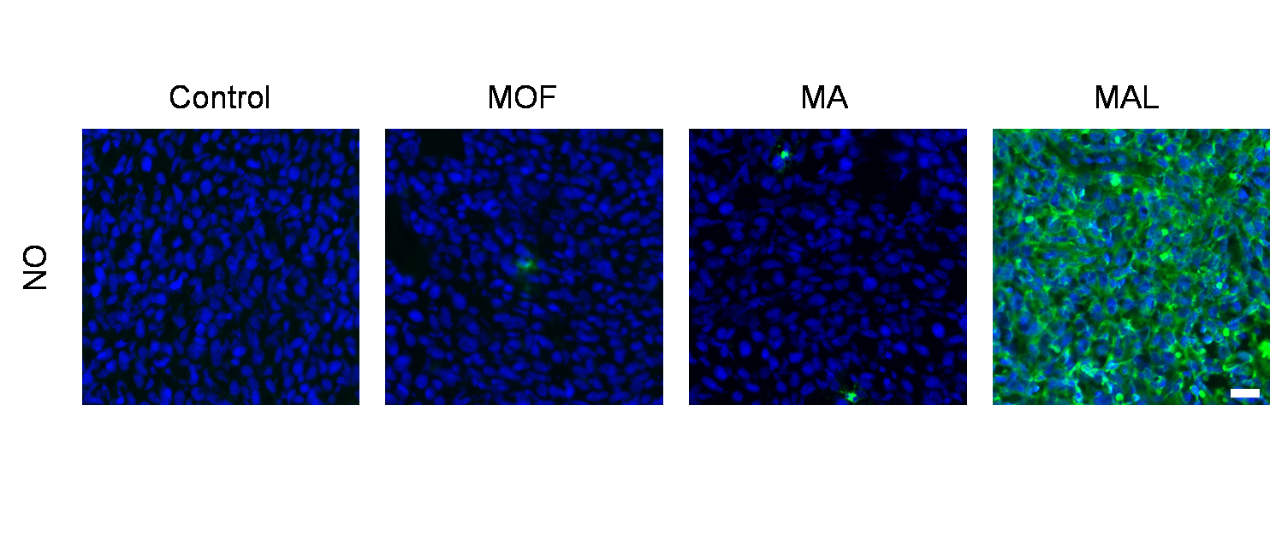


Figure S24: Representative NO staining images of tumor tissues after different treatments for 2 days. Scale bar: 20 μm.


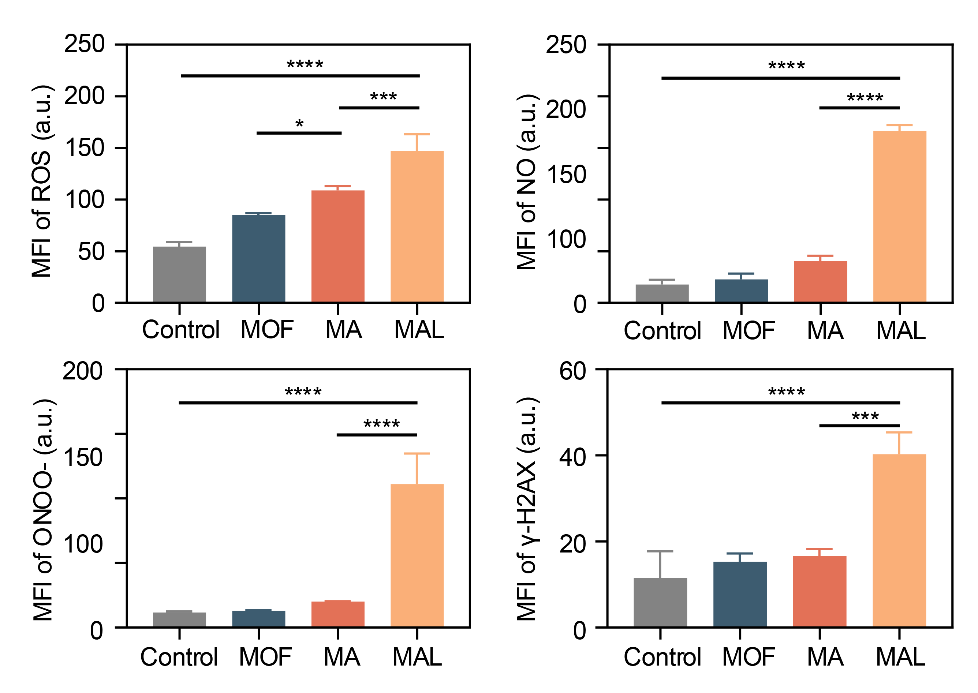


Figure S25: Fluorescence quantification of ROS, NO, ONOO^–^, and γ-H2AX.


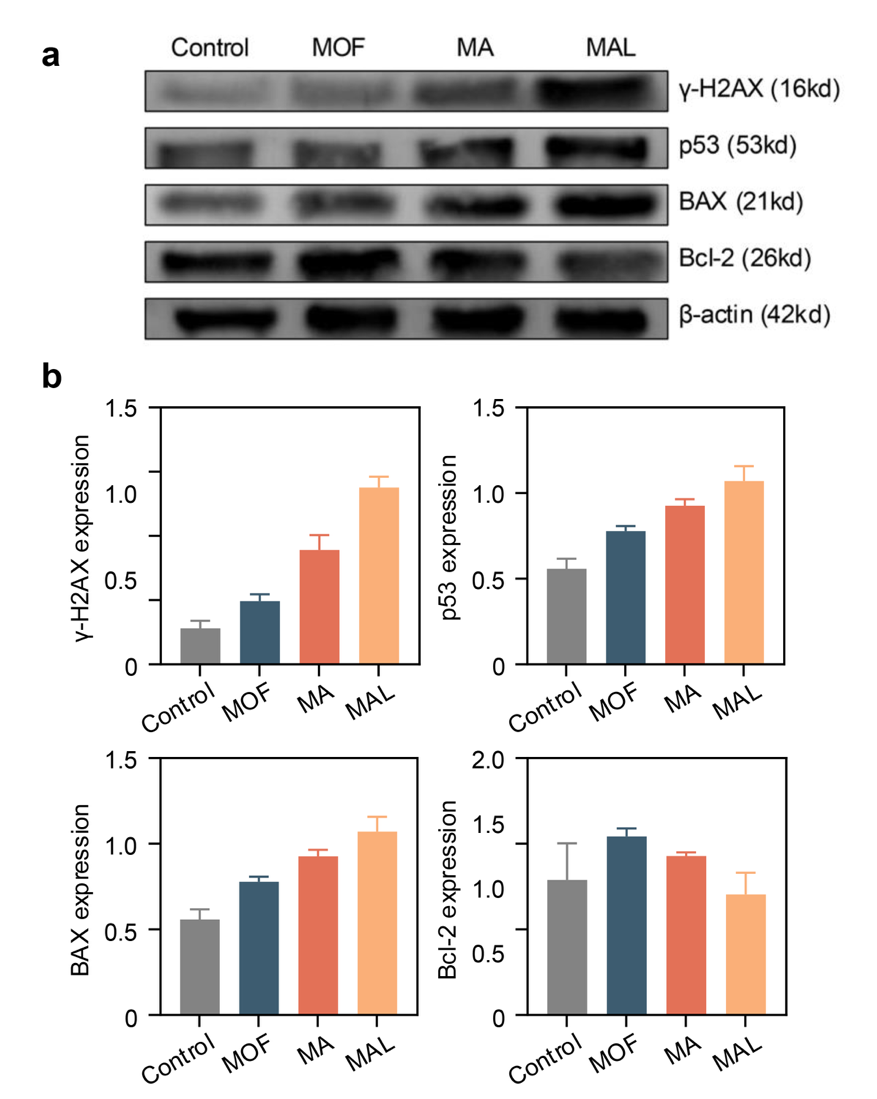


Figure S26: a) Western blotting assay of γ-H2AX, p53, BAX, and Bcl-2 in tumor. Samples derived from the same experiment and gels/ blots were processed in parallel. The experiment was repeated a third time independently with similar results. b) Western blot analysis of the expression levels of γ-H2AX, p53, BAX, and Bcl-2 in tumor incubated with different treatment.


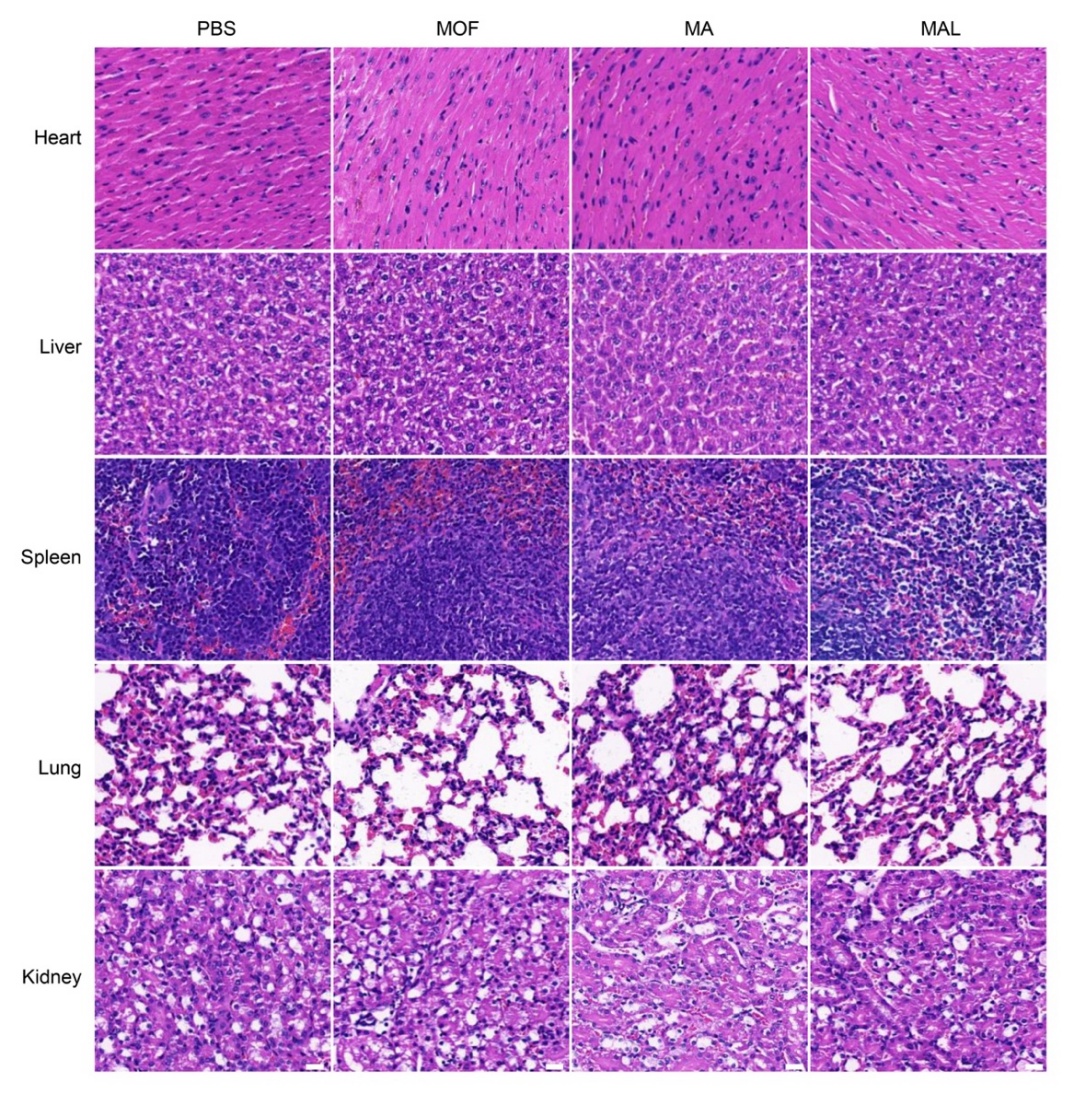


Figure S27: Hematoxylin and eosin staining of major organs (heart, liver, spleen, lung, and kidney). Scale bar: 20 μm.


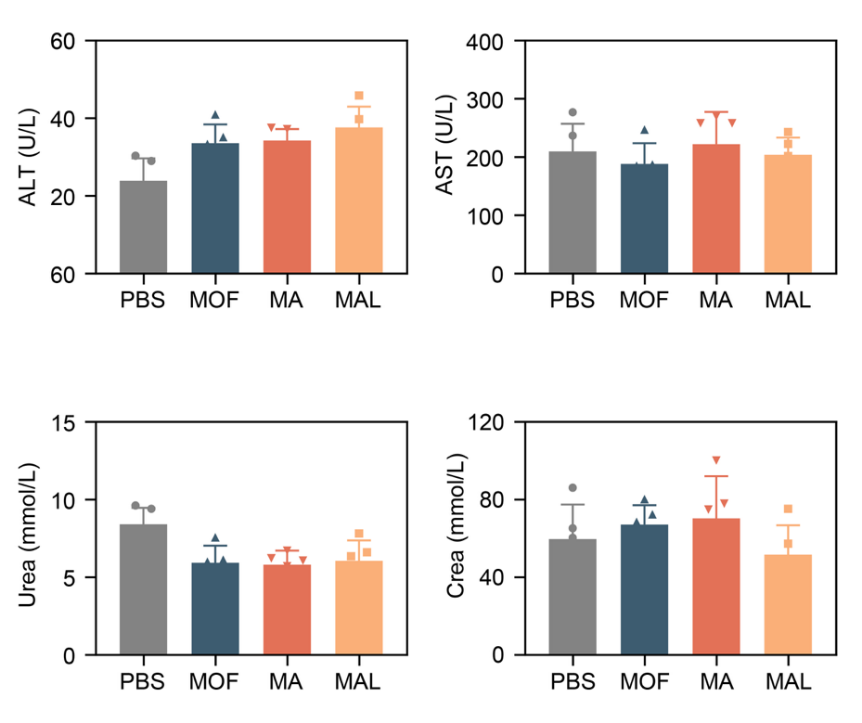


Figure S28: ALT, AST, Urea, and Crea levels of mice in different groups (n = 5). The

blood specimens were gathered when the animals were euthanized.


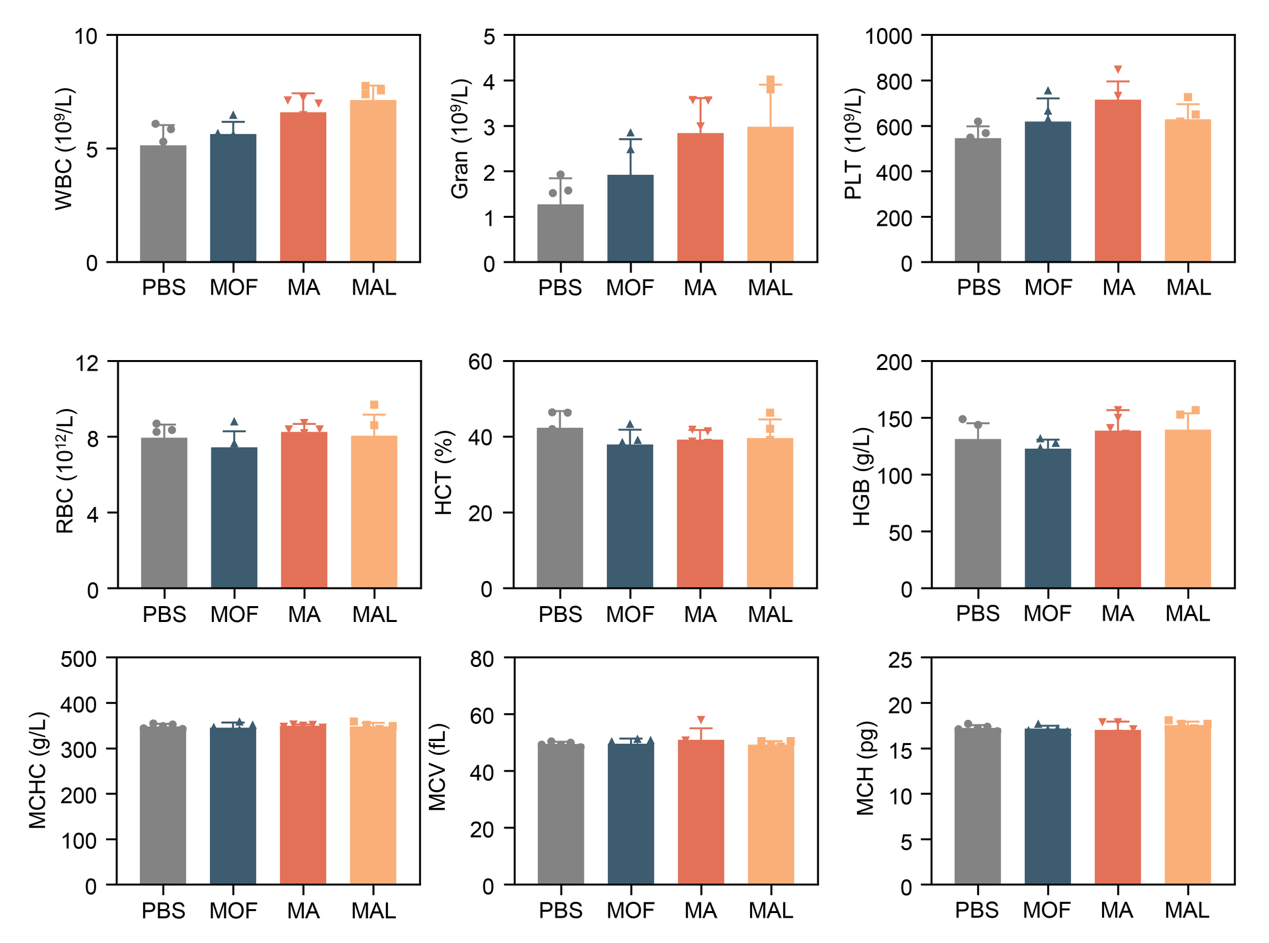


Figure S29: WBC, Gran, PLT, RBC, HCT, HGB, MCHC, MCV, and MCH levels of mice in different groups (n = 5). The blood specimens were gathered when the animals were euthanized.


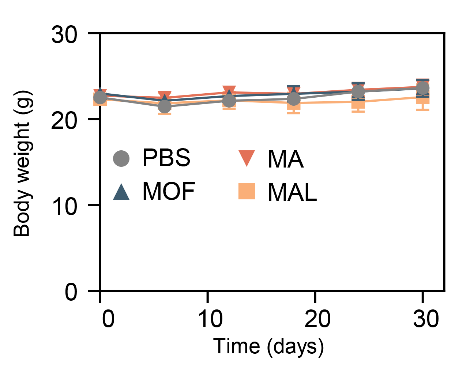


Figure S30: Body weight trends of mice after treatment (n = 5).


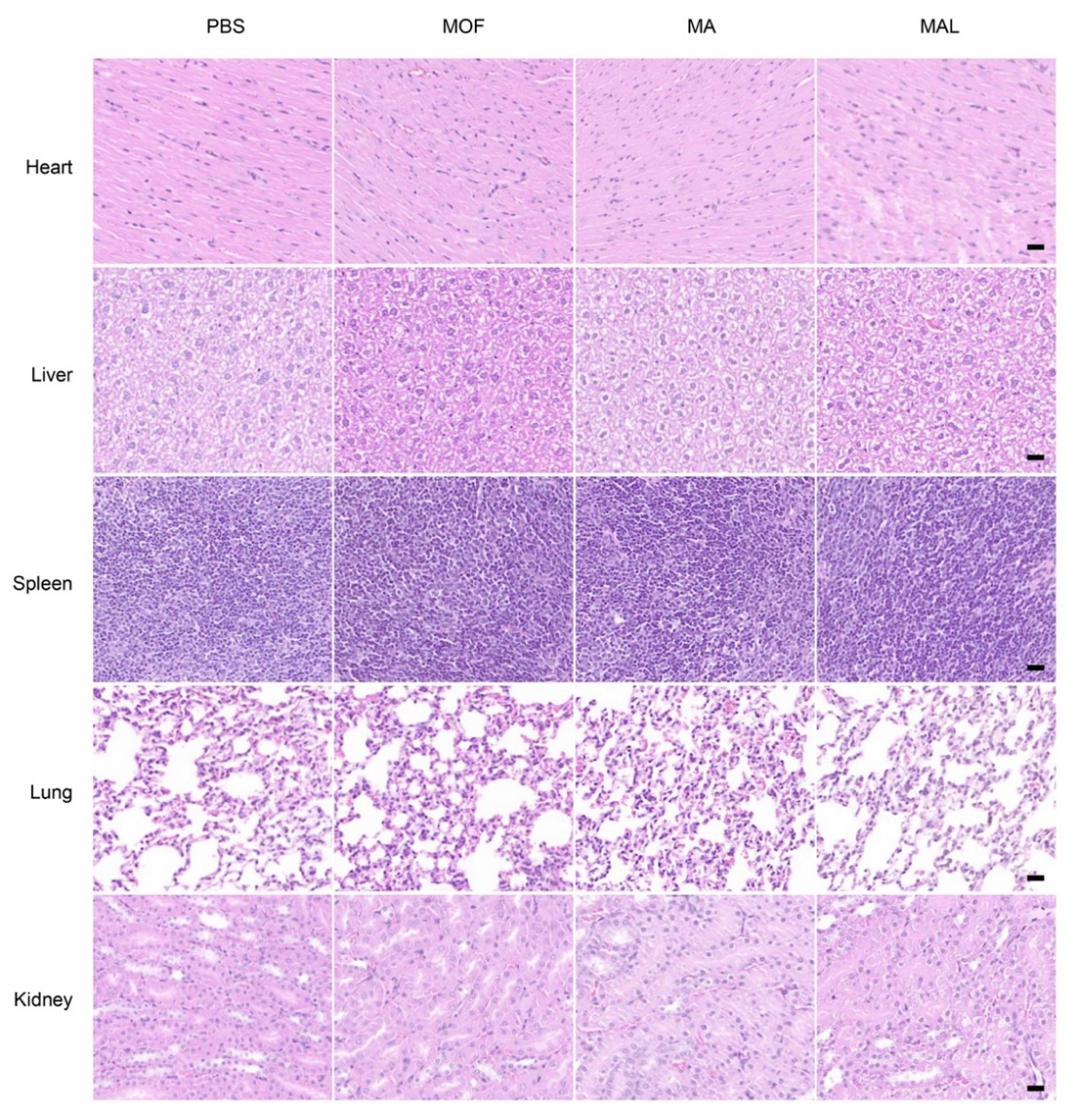


Figure S31: Hematoxylin and eosin staining of major organs (heart, liver, spleen, lung, and kidney) after 30 days of treatment. Scale bar: 20 μm.


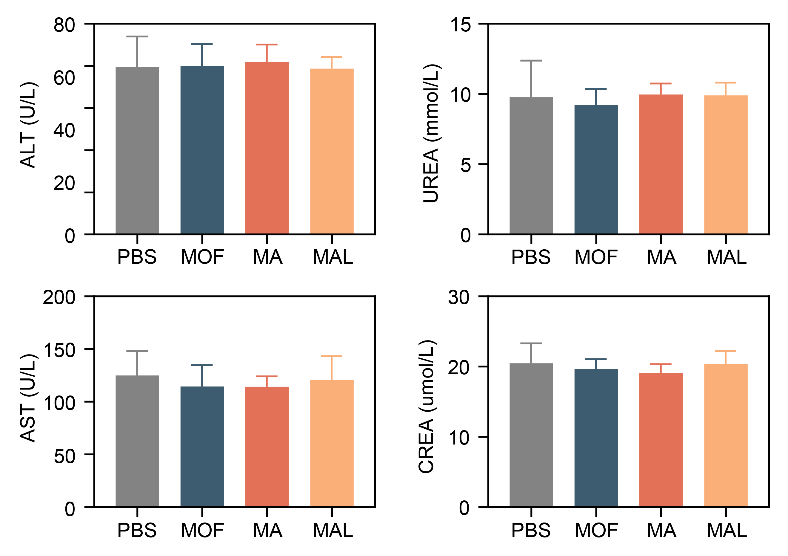


Figure S32: ALT, AST, Urea, and Crea levels of mice in different groups after 30 days of treatment. (n = 5). The blood specimens were gathered when the animals were euthanized.


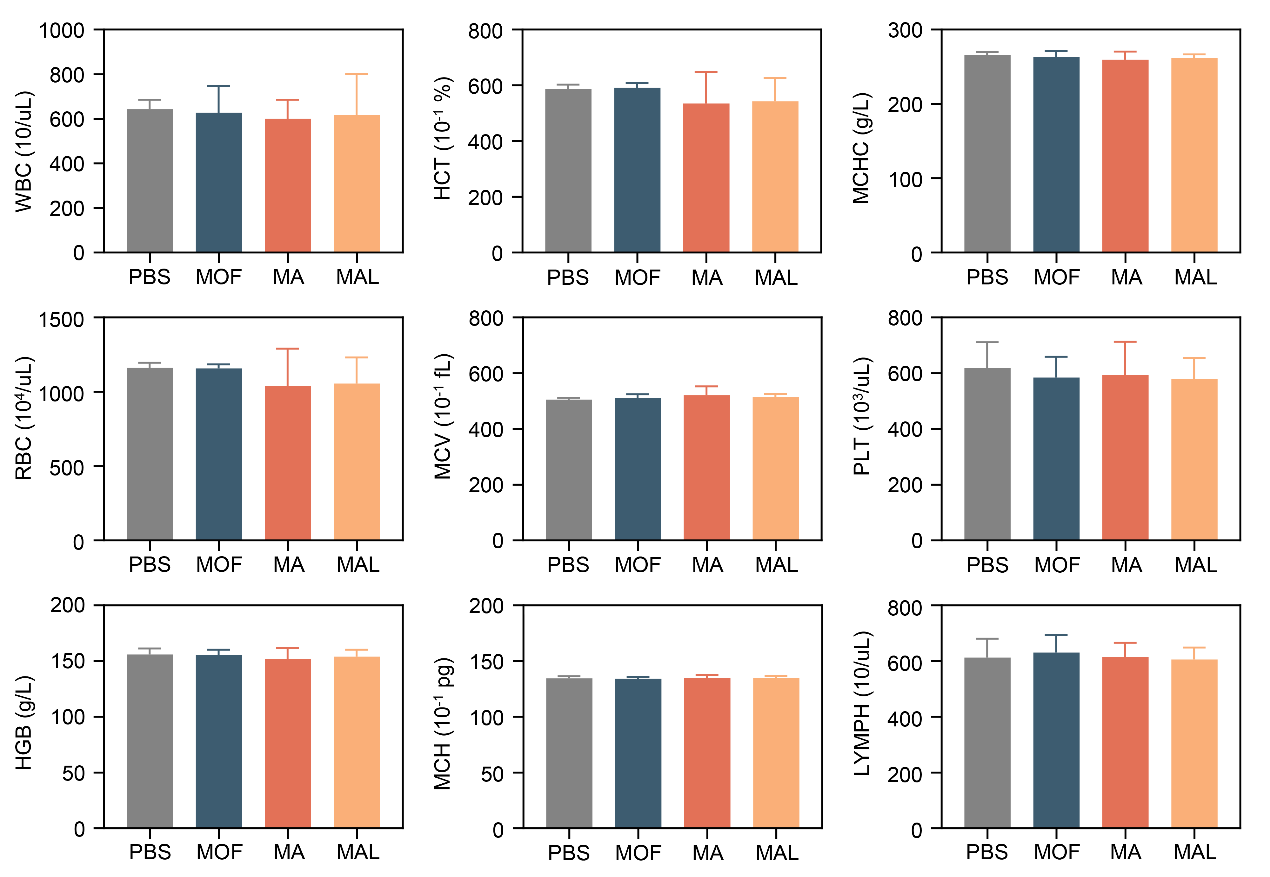


Figure S33: WBC, HCT, MCHC, RBC, MCV, PLT, HGB, MCH, and LYMPH levels of mice in different groups (n = 5). The blood specimens were gathered when the animals were euthanized.
